# Towards fully automated inner ear analysis with deep-learning-based joint segmentation and landmark detection framework

**DOI:** 10.1038/s41598-023-45466-9

**Published:** 2023-11-04

**Authors:** Jannik Stebani, Martin Blaimer, Simon Zabler, Tilmann Neun, Daniël M. Pelt, Kristen Rak

**Affiliations:** 1 Magnetic Resonance and X-Ray Imaging Department, Fraunhofer Institute for Integrated Circuits IIS, 97074 Würzburg, Germany; 2 Universität Würzburg, Experimentelle Physik V, 97074 Würzburg, Germany; 3 Department of Oto-Rhino-Laryngology, Plastic, Aesthetic and Reconstructive Head and Neck Surgery and the Comprehensive Hearing Center, Universitätsklinikum Würzburg, 97080 Würzburg, Germany; 4 Faculty of Computer Science, Deggendorf Institute of Technology, Deggendorf, Germany; 5 Institute for Diagnostic and Interventional Neuroradiology, Universitätsklinikum Würzburg, 97080 Würzburg, Germany; 6 Leiden Institute of Advanced Computer Science (LIACS), Universiteit Leiden, Leiden, CA 2333 The Netherlands

**Keywords:** Software, Anatomy, Diagnosis, Medical imaging, Bone imaging, Three-dimensional imaging, Tomography

## Abstract

Automated analysis of the inner ear anatomy in radiological data instead of time-consuming manual assessment is a worthwhile goal that could facilitate preoperative planning and clinical research. We propose a framework encompassing joint semantic segmentation of the inner ear and anatomical landmark detection of helicotrema, oval and round window. A fully automated pipeline with a single, dual-headed volumetric 3D U-Net was implemented, trained and evaluated using manually labeled in-house datasets from cadaveric specimen ($N=43$) and clinical practice ($N=9$). The model robustness was further evaluated on three independent open-source datasets ($N = 23{} + 7{} + 17$ scans) consisting of cadaveric specimen scans. For the in-house datasets, Dice scores of $\text{0.97 and 0.94}$, intersection-over-union scores of $\text{0.94 and 0.89}$ and average Hausdorff distances of $0.065{}$ and $0.14{}$ voxel units were achieved. The landmark localization task was performed automatically with an average localization error of $\text{3.3 and 5.2}$ voxel units. A robust, albeit reduced performance could be attained for the catalogue of three open-source datasets. Results of the ablation studies with 43 mono-parametric variations of the basal architecture and training protocol provided task-optimal parameters for both categories. Ablation studies against single-task variants of the basal architecture showed a clear performance benefit of coupling landmark localization with segmentation and a dataset-dependent performance impact on segmentation ability.

## Introduction

The benefit of cochlear implantation has been shown in various studies and reviews^1^. For a safe cochlear implantation base, it is essential to have a realistic pre- and intra-operative anatomical information about the cochlea and adjacent inner ear structures, not only to select the correct electrode for obtaining an appropriate cochlear coverage^2,3^, but to also prevent damage to intracochlear structures^4^. Furthermore, anatomical information about the inner ear may be used to elucidate the pathophysiology of hearing loss and balance disease in large scale evaluations of radiological data. Non-invasive radiological imaging of the temporal bone via computed tomography (CT) or magnetic resonance imaging (MRI) is currently the default strategy to provide the required anatomical information to the researcher or clinician. Inside the current routine workflow, the data is inspected visually as a series of 2D multiplanar views to mentally comprehend the 3D structure and gather the desired information. The automated creation of advanced 3D visualizations on the basis of semantic segmentation in conjunction with parameter estimation can thus be viewed as highly beneficial to the process of cochlear implantation in particular^5^ and inner ear clinical research in general. However, the generation of accurate and individualized analysis from radiological data remains time, cost and labour intensive. The generation process is bottlenecked by the step of manual labeling key structures due to the complexity and subtleties of the involved anatomy^6^ and suffers from inter- and intra-observer variability. To realize the envisioned benefits for clinical purposes and fix the pitfalls of the manual workflow, the two related tasks of a) semantic segmentation of inner ear structures and b) localization of landmarks have to be solved by a fully automated pipeline with minimal user interaction. Success in these two fundamental tasks may act as a gateway to fully automated cochlear duct length measurements, supplanting current formula-based methods relying on Bézier curves fitted through manually set keypoints^7^. To approach the automatization goal, we consider recent progress in the field of computer vision (CV) that has been spurred disruptively by deep learning with convolutional neural networks^8^ as the architecture of choice. Achieving state-of-the-art performance in image classification^9^ and medical image analysis^10^ tasks alike, they can drastically simplify them via automation by learning from a pre-assembled set of training examples. Careful compilation of the training examples enables the minimization of intra- and inter-observer variability. In contrast to more traditional machine learning algorithms, deep neural nets are regarded as adequate for operating with high-dimensional, unstructured data like volume CT data due to their capacity to internally construct salient data features during the optimization phase. An integrated, performant and data-efficient deep learning framework for automated analysis of inner ear structures could be beneficial for fast preoperative planning and large-scale clinical research.

### Related work

As a starting point in related literature, traditional methods like atlas-based frameworks^11^ for cochlear structure segmentation and inner ear fluid space^12^ or iterative random-walk with shape priors for full inner ear segmentation in $\mathrm {\mu }$CT data^13^ have been suggested in the past. Within the realm of deep learning based methods, there exist several closely related and recent studies that directly share the goal of automated analysis of inner ear structures from volumetric CT data. In the following, we provide a summary of these adjacent works.

In Heutink et al.^14^, sequentially staged 2D ResNet and 2D UNet were used to segment the cochlea from 3D ultra-high resolution clinical CT data in the context of automated cochlea length and volume measurement. The proposed system was formulated with 123 volume CT scans. Here, the 2D to 3D gap was bridged by using a 2D patch sampling approach that was iteratively integrated into volumes. The protocol leveraged the primary ResNet stage to generate soft-masking probability maps on three different scales with differing networks. The soft masks were then utilized to constrain the outputs of the secondary UNet stage to the previously identified foreground region. The augmentation protocol for the patches comprised in-plane rotations with an amplitude of $\pm {20}^{\circ }$ and vertical flips. The authors describe that validation was performed on a fixed set of eight volumes and testing on a fixed set of 75 volumes. No cross validation (CV) strategy is described.

Another adjacent study by Neves et al.^15^ employed a set of 3D CNN variants to perform multi-class segmentation on 150 clinical volume CT temporal bone scans. Three backbones, ResNet, UNet and the newly proposed AHNet were compared. The framework was presented as an integrated pipeline in the 3DSlicer software^16^ that makes use of largest region extraction and growing/shrinking algorithms with pre-set intensity values to postprocess all convolutional neural network outputs. The augmentation protocol encompassed intensity oscillations (10% of intensity magnitude) and flipping about the first axis. The validation and testing procedure for this work encompassed five-fold CV and a separate testing set of 25 volumes.

The work by Hussain et al.^17^ leveraged open-source micro CT data of 17 cadaveric specimen from Gerber et al.^18^ to formulate a 2D, multi-aspect CNN-based framework for inner ear segmentation. The authors introduced this as AutoCasNet, a three-stage cascade of networks specialized on the iterative segmentation of coronal, axial and sagittal aspects of the data, respectively. The authors operated and compared ResNet, UNet and SEUNet (Squeeze-and-Excitation Network,^19^) as backbones. No other design choices were tested, while validation and testing was done via 4-fold CV without an independent test set. The formulation incorporated largest region extraction to postprocess the network predictions. No data augmentation was described for this formulation.

Fauser et al.^20^ presented a shape-regularized preoperative pipeline that segments multiple anatomical risk structures from volumetric temporal bone scans to facilitate subsequent trajectory planning. In their first study, a triplet of multi-aspect 2D UNets, similar to the approach by Hussain et al., was used to segment the structures. In contrast to Hussain et al., who adopted AutoCasNet as a sequential cascade of convolutional neural networks, they made use of parallel inference for the axial, sagittal and coronal aspect-wise UNet instances and combined the result using a majority-voting strategy. Follow-up work^21^ replaced the parallelized multi-aspect set of 2D UNets with a two-staged 3D UNet implementation. There, the primary stage extracted a volume of interest, from which the secondary stage 3D UNet with identical architecture produced the segmentation of the anatomy. Both described systems shared key features. They exploited registration to probabilistic active shape models (PASM) as a separate downstream postprocessing step after network predictions. Further, both were formulated using data from 24 clinical volume CT scans without data augmentation. The validation procedure consisted of a two-fold cross validation strategy without an independent test set.

A summarizing table of all related works concerned with deep learning based automated analysis of temporal bone scans is provided by Table 1.

In addition to the presented works operating with CT imaging modality, further studies with volumetric magnetic resonance imaging (MRI) data were conducted recently. We refer to two studies that engaged with inner ear segmentation based on MR data generated by T2-weighted sequences. Notably, Vaidyanathan et al.^22^ described a pipeline based on a modified 3D UNet, achieving an inner ear segmentation Dice score of 0.86(1) (mean and standard deviation (SD)) for the validation dataset and 0.87(1) for the test dataset. Ahmadi et al.^23^ employed a 3D V-Net^24^ based pipeline in conjunction with an atlas-based pre-localization and pre-segmentation step to achieve a Dice score of 0.90(2) for the test dataset. These presented studies are concerned with segmentation tasks in a mono- or multi-class setting.

This work, however, proposed the segmentation and landmark detection as an integrated multi-task problem, since a single, bi-headed network performs both segmentation and landmark detection using a multi-task learning approach. Multi-task learning can produce improved results compared with learning each task separately^25^ and has been applied successfully in a large variety of tasks in medical imaging^26^, including brain image analysis^27^ and retinal image analysis^28^. For inner ear analysis, multi-task learning was used for reducing metal artifacts in post-implantation CT images^29^. To further contextualize the presented work with multi-task joint segmentation and landmark detection, we look to craniomaxillofacial (CMF) applications, since for inner ear analysis and otological applications, multi-task learning has not been applied extensively. For example, Liu et al.^30^ presented a joint segmentation and landmark detection framework for CMF preoperative planning. The automated task comprised the segmentation of midface and mandible bone structures and the localization of 175 bone, teeth and face landmarks. The design reinterpreted landmark localization as multi-class segmentation by casting landmarks as individual classes in the spirit of semantic segmentation. The proposed framework leveraged a layered setup of a quadruplet of specialized 3D UNets operating on different image resolution scales. A primary stage provided a coarse segmentation and coarse-global localization of bony and facial landmarks only, while a secondary stage comprised two refinement models that handled bone segmentation and landmark detection separately. The secondary stage then produced refined segmentations and localization results, using specialized bone and tooth models from the purpose-cropped volume of interest inputs of the primary stage.

Zhang et al.^31^ proposed a context-guided UNet based framework termed JSDNet for joint midface and mandible bone segmentation and localization of 15 landmarks for CMF applications. The architecture consisted of a cascade of two sub-networks, subsequently termed FC1 and FC2. The first network was trained to estimate displacement maps from landmark positions, while the second network conducted the joint prediction of the bone segmentation maps and landmark heatmaps using CT scan data concatenated with displacement map data as inputs.

### Aims of the study

The main purpose of our project was the development and validation of a fully automated pipeline to jointly generate segmentation maps of inner ear structures and the localization of landmarks from temporal bone CT scans. By framing the problem as a multi-task learning problem, we implemented an end-to-end trained solution algorithm based on the U-Net architecture. In this work, we provide contributions in the following aspects: (1) describing a performant and end-to-end differentiable U-Net pipeline that is evaluated against a range of in-house and open source data from clinical and specimen origin, (2) directly comparing the formulation with single-task UNets, (3) comparing the formulation to recently published state-of-the-art methods with temporal bone analysis applications and to our reimplementation of a joint segmentation and localization architecture from the craniomaxillofacial application space, (4) providing a series of ablation studies elucidating the performance impact of various design choices and (5) releasing the labeled training and validation dataset. The existence of labeled data is vital to the principally data-scarce and restricted field of medical imaging, making the open-source release of the labeled in-house datasets on the Zenodo platform (DOI: https://doi.org/10.5281/zenodo.8277159) the last contribution element.

**Table 1:** Overview comparison of recently published state-of-the-art methods for machine-learning based inner ear segmentation.

Reference	Data	Voxel dims	Splits	Architecture	Tasks	Dice score
Heutink et al.^14^	Clinical, internal	{45${\upmu }$m, 50${\upmu }$m}	(40, 8, 75, –)	Sequentially-staged 2D ResNet for soft masking and 2D UNet for segmentation	Core cochlea segmentation	0.90(3)
Neves et al.^15^	Clinical, internal	{125${\upmu }$m, 250${\upmu }$m}	(125, 25, –, 5)	AH-Net based on 3D UNet, postprocessing via largest region extraction and growing/shrinking based on preset intensity values	Multi-class segmentation	ResNet 0.91(3), UNet 0.89(4), AHNet 0.88(4)$^{\dagger }$
Hussain et al.^17^	Cadaveric, open	{16.3${\upmu }$m, 19.5${\upmu }$m}	(17, *, –, 4)	AutoCasNet: sequential 2D multi-aspect cascade with UNet, ResNet and SEUNet backbones	Inner ear segmentation	0.90(7)$^{\ddagger }$
Fauser19 et al.^20^	Clinical, internal	$200 \times 200 \times 400$ ${\upmu }\textrm{m}^{3}$	(24, 12, –, 2)	Parallel 2D multi-aspect UNet with majority voting and probabilistic active shape model postprocessing	Multi-class segmentation	0.85(7)$^{\ddagger }$(Cochlea) 0.80(7)$^{\ddagger }$ (SSC)
Fauser20 et al.^21^	Clinical, internal	$200 \times 200 \times 400$ ${\upmu }\textrm{m}^{3}$	(24, 12, –, 2)	Two sequentially-staged 3D UNet: VOI extraction and subsequent segmentation, postprocessing via probabilistic active shape models	Multi-class segmentation	0.87(3)$^{\ddagger }$ (Cochlea) 0.85(3)$^{\ddagger }$ (SSC)
Nikan et al.^32^	Cadaveric, internal	{154${\upmu }$m, 625${\upmu }$m, 100${\upmu }$m}	(126, 14, 18, –)$^{+}$	3D UNet with balanced window sampling and augmentation layers	Multi-class segmentation	0.90(–)
current work	Clinical, cadaveric, internal, open	99${\upmu }$m	(38, 5, 56, 3)	3D AttentionUNet, test-time augmentation	Joint inner ear segmentation and landmark localization	0.94(1) (test set B) 0.92(3) (all)

The splits define the number of training, validation and test dataset instances ($N_{\textrm{train}}$, $N_{\textrm{val}}$, $N_{\textrm{test}}$, $N_{\textrm{CV}}$) as well as the number of cross validation folds. For the works with multi-class segmentation tasks, the inner ear was one of the categories or could be recovered as a composite of multiple categories. VOI denotes *volume of interest*. A single length value denotes isotropic voxel dimensions. Curly brackets indicate a set of multiple base voxel dimensions. $^{*}$Hussain et al. utilized four fold CV but did not specify the size of the fold. $^{+}$Nikan et al. utilized fixed augmentation layers to increase the data by simulating blurred and resampled micro CT data and clinical CT data. The original instances were (31, 4, 18) for training, validation and test. †The indicated scores were given for the direct network output. After postprocessing, the scores equally amounted to 0.91. $^{\ddagger }$ Generated on CV-based validation without independent test set.

## Methods

### Datasets description

Design, training and evaluation was performed on the basis of two in-house datasets. Both were scanned on a Siemens Axiom-Artis DynaCT fpVCT scanner (Siemens Healthcare GmbH, Erlangen, Germany) using commercially available software (Syngo DynaCT, Siemens). The following acquisition parameters were utilized: 20s DCT Head protocol, tube voltage 109 kV, tube current 21 mA, pulse length 3.5 ms, rotation angle 200$^{\circ }$, frame angulation step 0.5$^{\circ }$ per frame, slice thickness 466 $\upmu$m. In the postprocessing step, the acquired data was reconstructed with a HU kernel type with sharp image characteristics and was exported to the Digital Imaging and Communications in Medicine (DICOM) file format^33^. Finally, this acquisition and postprocessing protocol yields cuboidal voxels with an isotropic edge length of $a_{\textrm{v}} = {98.6}{\upmu }\textrm{m}$. The first in-house *Dataset A* (*ex-vivo*) was comprised of scans of 43 cadaveric temporal bone specimen and was split into two subsets for training ($N = 38$) and validation purposes ($N = 5$). The second in-house *Dataset B* (*in-vivo*) was comprised of $N = 10{}$ additional temporal bone scans that were recorded *in-vivo* from pre-operative patients from clinical practice. The data for the clinical dataset B were processed in an entirely anonymized and retrospective fashion. The retrospective anonymized study was conducted in concordance with local guidelines and principles of the Declaration of Helsinki and Good Clinical Practice^34^ and was approved of the Ethics committee of the University of Würzburg (2019020401). The requirement for informed consent from the study subjects was waived by the Ethics committee of the University of Würzburg due the anonymous data analysis and Article 27, Paragraph 4.1 of the Bavarian hospital law (BayKrG), which permits the use of patient data within the hospital for research purposes or in the research interest of the hospital without the patient’s consent being necessary. The instances of dataset *B* were exclusively used as a hold-out set and main gauge for the performance of the methodology. From the total 10 dataset elements, a single instance showed otosclerotic lesions and was evaluated separately. This fact is communicated through the 9 + 1 element notation for dataset B. For the described datasets A and B, the inner ear structure was manually annotated under the supervision of a cochlear implant surgeon (K.R.) with extensive experience in cochlear analysis. The segmentation and landmark position annotation was performed with the 3DSlicer software^16,35^, an open-source software tool for medical image analysis. The segmentation process comprised manual labeling via brush-based painting assisted by masks generated via voxel intensity thresholding. The foreground labels thus correspond to the inner ear or labyrinth fluid space that is bounded by bone and the membranes round and oval window. As anatomical landmark labels, the positions of helicotrema, oval window and round window were marked as a voxel coordinate vectors. Dataset A is released as open-source at the Zenodo repository (DOI: 10.5281/zenodo.8277159) as part of this work.

**Figure 1:**
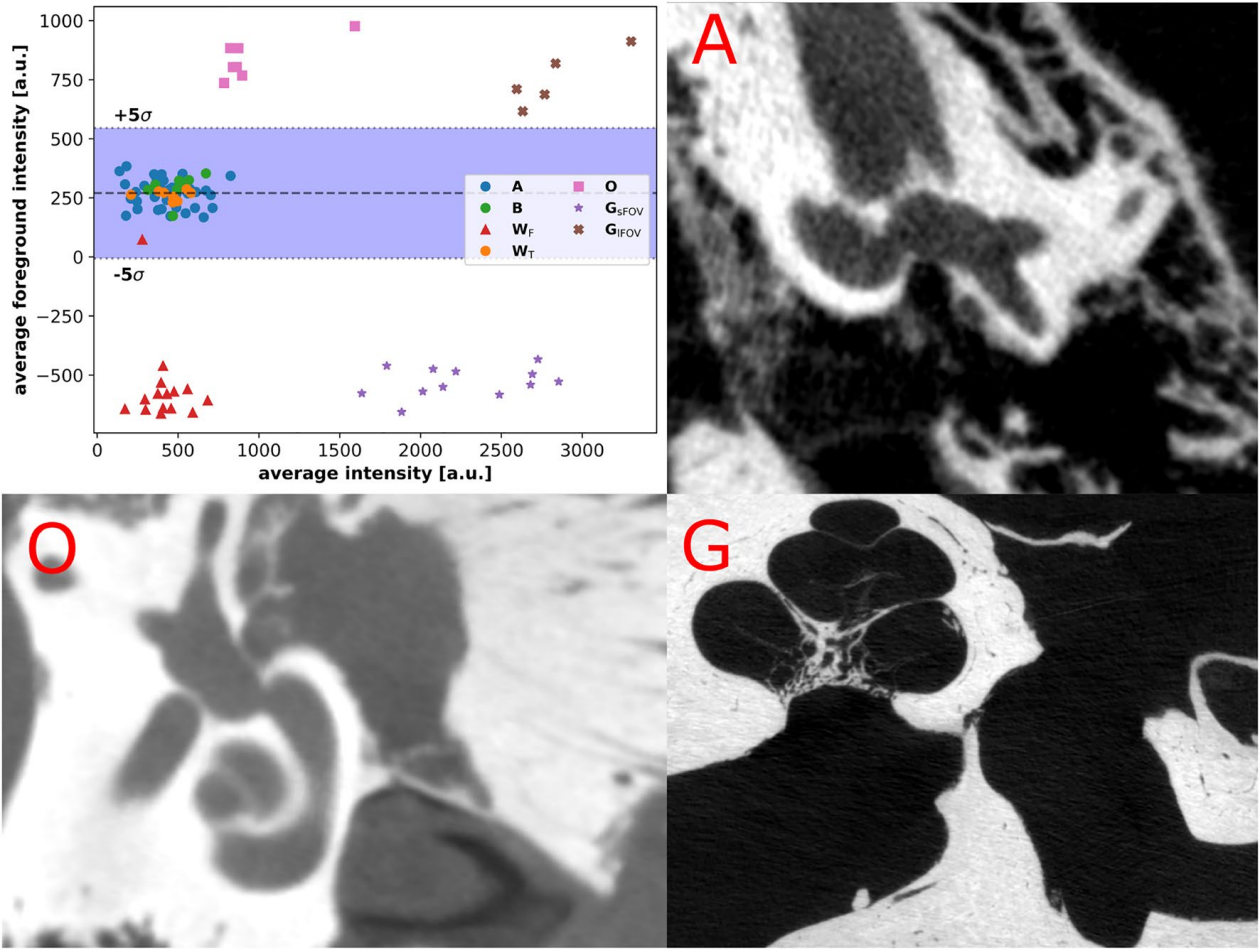
Visual rendition of the contrast distribution of the utilized datasets. Processing for the open-source datasets was performed on the four identifiable clusters separately. The shaded blue region demarcates the five standard deviation region of average foreground voxel intensity of the training dataset. The dashed line marks the corresponding average foreground voxel intensity. Upper right panel A shows an exemplary slice of the training dataset, lower left panel shows a slice of the embedded specimen dataset O and lower right panel shows a slice of an instance of microCT dataset G.

In order to further examine the performance and generalization ability of the proposed methodology, evaluations with three publicly available, open source CT datasets were conducted. These datasets presented a challenge to the machine learning model due to significant differences in imaging setup, native voxel sizes and annotation sources. All open-source datasets provided similar inner-ear labels for segmentation purposes. Missing landmark position information in two of the open source datasets was supplemented by the authors using the same 3DSlicer based, voxel coordinate based workflow used for the landmark annotation of the in-house datasets. The open access *Dataset W* by Wimmer et al.^36^ consists of $N=23{}$ specimens that were imaged with a clinical multi-slice CT (MSCT) device, yielding a voxel size ($150 \times 150 \times {200}{\upmu }{\textrm{m}}^{3}$). The specimen were preserved by embalming them with Formalin and Thiel, yielding two distinct subgroups F and T, respectively. For the Formalin subgroup, the embalming process altered the contrast of usually fluid-filled volumes in comparison to other anatomy, see Fig. 1. The OpenEar *Dataset O* by Sieber et al.^37^ contains $N=7{}$ temporal bone scans from the cone-beam CT (CBCT) modality and an isotropic voxel size of $125 \times 125 \times {125}{\upmu }{\textrm{m}}^{3}$. The preservation method of the specimen entailed multiple preservation steps of successive immersion in five different fixation solutions and physical access canal drilling to the superior semi-circular canal. The recorded specimen were embedded in epoxy resin as a final step. This procedure also introduced contrast-shifts due to modification of fluid-filled volumes as well as structural modification of bone anatomy. The high-resolution *Dataset G* by Gerber et al.^18^ provides $N=17$ temporal bone scans using microCT modality and isotropic voxel sizes of $\{{16.3}{\upmu }{\textrm{m}}, {19.3}{\upmu }{\textrm{m}}\}$. This dataset also has two distinct subgroups with larger and smaller field-of-view with respect to the inner ear anatomy. The contrast distribution of the two subgroups is well-differentiable as visible in Fig. 1. This dataset generally contains dry specimen that do not provide contrast between *in-vivo* fluid-filled volumes and other structures. The preparation method also erased the round and oval window membranes (see Fig. 1 panel G). The imaging and reconstruction procedure for all open-source datasets appears to have deviated from the standard operating procedure for clinical imaging. This implies that the orientation of the open-source datasets in the volume differs from the predominant orientation visible in the in-house datasets A and B. We visualized this phenomenon in the accompanying Binder notebook online resource https://github.com/stebix/cochlea-spatial-investigation. For the presented deep learning algorithm, the manually produced segmentation labels were considered as the basal ground truth. The segmentation labels were provided as integer per-voxel labels that signify the membership of the voxel to a salient anatomical structure, while ground truth landmark labels are given by their manually determined voxel coordinate vectors. Summarizing information about the features of the different datasets is displayed in Table 2. A visual comparison of the various datasets, comprising preparation-induced contrast differences and imaging induced voxel size differences is provided in the supplementary material Sect. S.4.

**Table 2:** Overview of the defining dataset parameters utilized for the development and evaluation of the proposed pipeline.

ID	Source	Purpose	Availability	Voxel size	*N*	Labels
A	Frozen specimen	Train/Val	Open	${99}{\upmu \textrm{m}}$	43	SM, LM
B	Clinical	Test	Internal	${99}{\upmu \textrm{m}}$	9 + 1	SM, LM
W	Embalmed specimen	Test	Open	$150 \times 150 \times 150$ $\upmu \textrm{m}^3$	23	–
O	Embedded specimen	Test	Open	$125 \times 125 \times 125$ $\upmu \textrm{m}^3$	7	LM
G	Dry specimen	Test	Open	$\{{16.3}{\upmu \textrm{m}}, {19.5}{\upmu \textrm{m}}\}$	17	LM

For the labels column, SM and LM indicate segmentation maps and landmark positions that were newly generated for this study. For open-source datasets O and G, landmark labels were generated to supplement the raw and segmentation label data.

### Data processing and augmentation

The convolutional U-Net architecture is unaware of pattern scale anisotropy induced by varying voxel edge lengths. Thus, the datasets were globally resampled to the isotropic voxel size $a_v = {99}{\upmu }\textrm{m}$ of the training dataset A prior to inference.

For the in-house fpVCT data, we first clipped at the $0.01$-th and $0.99$-th quantile value and subsequently rescaled voxel intensity values such that a mean $\upmu = 0$ and unit standard deviation $\sigma = 1$ normal distribution ${\mathscr {N}}(0, 1)$ emerges for the full datasets. The parameter values for quantile *q*, mean $\upmu$ and standard deviation $\sigma$ were computed on the whole datasets respectively.

For the open-source datasets, we integrated a histogram and contrast matching scheme in addition to the above clipping and rescaling computation. This was a key aspect to correct for the fluid-space-wise contrast modifications introduced by the various preservation schemes of the datasets W (Formalin embalming), O (immersion in fixation solutions, resin embedding) and G (drying). Dataset A from frozen specimen exhibited well-preserved contrast for normally fluid-filled cavernous structures and membrane regions like the oval and round window compared to our clinical dataset. Another open-source dataset processing element was the application of the clipping and normalization procedure separately for each cluster visible in Fig. 1. The process yielded a remapping of the intensity distribution into the range of the training data, subsequently diminishing the input data domain shift algorithmically. For training and inference, all volume instances were divided into sub-volumes (termed *chunks*) of dimension $(128 \times 128 \times 128)$. Individual chunks were loaded and processed separately by the network. This chunk-based processing strategy attenuates the large memory consumption of volumetric data and 3D convolution operations. The chunks were generated in a sliding-window fashion with an isotropic stride of 25 voxels in *x*, *y* and *z* direction.

To enhance both effective training data amount and variability, the traditional form of training time augmentation was performed by stochastically applying a variety of augmenting transformations to a chunk. We used both spatially and intensity-wise transformations in our protocol. For the intensity-wise augmentation, we randomly adjusted image contrast by multiplication with a scaling factor and by application of additive Gaussian or Poisson noise. Lastly, a free angle rotation of the data about a randomly chosen spatial axis was performed. During our ablation studies, we employed two different variations of the protocol. For augmentation protocol Aug$_\alpha$, only fixed 90$^{\circ }$, 180$^{\circ }$ or 270$^{\circ }$ rotations about the first volume axis as well as free angle rotations with a amplitude of 30$^{\circ }$ were employed. The second augmentation protocol $\beta$ encompassed the fixed rotations about all three spatial axes and free angle rotations with a larger amplitude of 45$^{\circ }$. More information and visualizations concerning the augmenting transformations are provided in the supplementary materials. Besides training data augmentation, we applied test-time data augmentation during all inference runs of the external open-source datasets to mitigate the domain shift induced by the non-standard rotation state. For that, we automated rotations of the input chunks with angles 90$^{\circ }$, 180$^{\circ }$ and 270$^{\circ }$ about all three spatial axes. The resulting $N_{\textrm{TTA}}= 10$ predictions were then averaged on the voxel level to produce a final prediction. Further details on the data processing, training and testing augmentations are stated in the supplementary information Sect. S.4.

### Algorithm and network architecture

**Figure 2:**
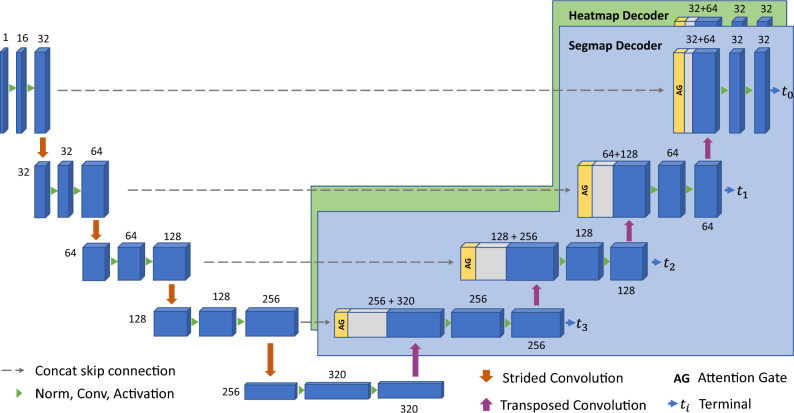
Overview block diagram of the encoder-decoder 3D U-Net architecture and deep supervision terminal structure that was implemented for the joint segmentation and landmark detection task. The U-Net architecture entails a single common encoder backbone and two task-specialized decoder heads. The cuboids symbolize feature maps produced by the convolution operation. The number of feature maps is stated for every convolution block operation next to the cuboid. The single encoder backbone hierarchically distills contextualized information from the input CT data while the two specialized decoder heads process the distilled feature maps to solve the semantic segmentation and heatmap regression task. In addition to the U-Net with the standard output terminal at the top-most decoder ($t_0$), a deep supervision strategy was adopted by producing secondary output maps at lower decoder levels (terminals $t_1$ to $t_3$). Attention gating at the skip connections was further used to filter feature maps for relevant foreground volume and improve overall performance.

For the inherently volumetric fpVCT data, the 3D U-Net architecture was shown^24,38^ to be an appropriate architecture for automated medical image analysis, outperforming many alternative techniques. Thusly, a fully convolutional and deeply supervised U-Net model was assembled to solve the joint segmentation and localization problem. Consisting of a common encoder block, two task-specific decoder blocks and skip inter-connections, the contracting encoder path hierarchically extracts high level information while decreasing spatial resolution through strided convolutions. For the dual task of segmentation and landmark localization, two separate decoder heads were envisioned that both draw upon the contextualized high level features extracted by the common encoder backbone. The expanding decoder path successively re-up-samples the feature maps and utilizes the extracted feature information, supplemented with localized details via the skip connections, to segment the voxels into semantic classes and localize landmark positions via heatmap regression, respectively. A visual overview of the utilized 3D U-Net architecture is provided in Fig. 2. Building upon heuristics derived from nnUNet^39^, a default network architecture with four levels and a double convolution module as the basic building block, consisting of a normalization layer, a $(3 \times 3 \times 3)$ convolution and a nonlinear activation function. In the spatially contracting encoder path, the downsampling operation is performed by strided convolutions with a kernel size of $(2 \times 2 \times 2)$ and isotropic stride 2. The feature map size or channel count cascade was given by the sequence $\{ 32, 64, 128, 256, 320 \}$ from higher to lower encoder levels. The number of feature maps was held constant during a level transition in both the encoder and decoder pathway. The upsampling operation in the spatially expanding decoder path was executed by a transposed convolution with a kernel size of $(2 \times 2 \times 2)$ and isotropic stride 2. This symmetric design enabled the direct concatenation of the feature maps produced by the left-adjacent encoder, further facilitating correct global localization of salient structures for segmentation and localization. The described encoder cascade yielded tensors of spatial dimension $\{ 64^3, 32^3, 16^3, 8^3 \}$ after every downsampling step.

The *segmentation head* performed the voxel-wise classification problem by estimating a pseudo-probability map $\textbf{S}\in \mathbb {R}^{n_x \times n_y \times n_z}$ that possesses identical shape with respect to the input. The final segmentation maps were obtained by applying a sigmoid nonlinearity to the last convolutional layer output. In the architecture sketch Fig. 2, the segmentation head is depicted by the light-blue colored structure. The *heatmap head* was the second specialized decoder structure that is attached to the common encoder backbone and performed the landmark localization task via the intermediate step of heatmap regression $\textbf{H}\in \mathbb {R}^{n_x \times n_y \times n_z}$. The heatmap head was structurally similar to the the segmentation head, but possessed three output channels to facilitate the localization of the landmarks helicotrema, oval and round window. Furthermore, the heatmap head did not process its last convolutional layer output with a sigmoid activation function. The coordinate predictions for a specific anatomical landmark were subsequently computed from the corresponding heatmap via localization of the mode through the argmax operation. Here, ${\textbf{r}}\in \mathbb {R}^3$ is the predicted coordinate vector and *k* denotes an index for the landmark, i.e. apex of the cochlea and oval window and round window. The heatmap head is depicted in the architecture diagram of Fig. 2 as the the green colored structure. Both decoder heads were attached to the common encoder backbone and received the feature maps from the lowest level of the encoder structure and successively upsampled them using their own set of internal weights and supplied skip connection inputs. The internal U-Net parameters were learned by minimizing the composite loss function given in Eq. (1), consisting of the segmentation-specific term ${\mathscr {L}}_{\textrm{segmentation}}$, the landmark-related and heatmap-specific term ${\mathscr {L}}_{\textrm{heatmap}}$ and the regularization term ${\mathscr {R}}({\textbf{w}})$. For the segmentation, the loss was computed as a linear combination of cross-entropy loss ${\mathscr {L}}_{\textrm{CE}}$ and Dice loss ${\mathscr {L}}_{\textrm{sDSC}}$. The Dice loss function was derived from the Dice-Soerensen similarity measure^40,41^.

1 $$\begin{aligned} {\mathscr {L}} = {\mathscr {L}}_{\textrm{segmentation}}+ {\mathscr {L}}_{\textrm{heatmap}}+ {\mathscr {R}}({\textbf{w}})= {\mathscr {L}}_{\textrm{CE}}+ {\mathscr {L}}_{\textrm{sDSC}}+ {\mathscr {L}}_{\textrm{MSE}}+ {\mathscr {R}}({\textbf{w}}) \end{aligned}$$

In the equation above the abbreviation CE denotes the cross entropy, sDSC denotes the (soft) Dice loss and MSE stands for mean squared error. The mathematical definitions for the utilized component loss function are described in detail the supplementary materials section S.6. The mean squared error loss was measured between the ground truth heatmap centered around the landmark coordinates and the predicted heatmap. The ground truth heatmap for a corresponding landmark coordinate vector from manual annotation was thereby produced by computing a 3D *Gaussian function* on the voxel grid with an amplitude $$\alpha$$ and a spread of $$\beta$$. To smoothly guide the network to learn accurate heatmaps in the voxel space, we utilized a heatmap scheduling protocol. In the protocol, we increased the amplitude parameter $$\alpha$$ and decreased the spread parameter $$\beta$$ according to a preset sequence of values at fixed training iteration numbers. Both loss components $${\mathscr {L}}_{\textrm{segmentation}}$$ and $${\mathscr {L}}_{\textrm{heatmap}}$$ were also utilized in the construction of the auxiliary or companion loss functions for the deep supervision terminals. The final regularization term $${\mathscr {R}}({\textbf{w}})$$ denotes the neural network regularization by imposing a zero-mean Gaussian prior over the weights. This yields the well-known weight decay functional $${\mathscr {R}}({\textbf{w}})= {\raise0.7ex\hbox{$1$} \!\mathord{\left/ {\vphantom {1 2}}\right.\kern-\nulldelimiterspace} \!\lower0.7ex\hbox{$2$}} \, \lambda \, {\textbf{w}}^2$$ embedded in the AdamW^42^ optimizer with the weight decay strength hyperparameter $$\lambda$$.

We performed a multitude of ablation experiments to elucidate the performance impact of various design choices. To that end, we defined a base architecture using the feature map cascade shown in Fig. 2 together with instance norm^43^, leaky ReLU nonlinearity with slope parameter 0.025, heatmap scheduling and combined Dice-Cross-Entropy-Loss with deep supervision. Starting from this fixed basal configuration, we mono-parametrically changed the following aspects: normalization layer, loss function, encoder depth and channel dimension size, augmentation strategy, activation function and attention gating (global and head-wise). For the normalization layer we tested against batch norm. For the loss function we tested the segmentation part against pure Dice loss, squared Dice Loss^24^, LogCoshDiceLoss^44^, pure binary cross entropy loss and against disabled deep supervision. For the training data augmentation scheme we tested against an enhanced scheme (Aug$_\beta$) and no augmentation at all. For the activation function we tested against ReLU, PReLU, GELU^45^, SiLU^46^ and Mish^47^. Further details about basal and ablation architectures are provided in the supplementary materials Sect. S.1. For attention gating, we tested against partial head-wise and global application of attention gating. Every configuration was trained with three-fold CV and tested with all hold out sets.

To provide a concrete baseline result against published framework, we turned towards the JSDNet described by Zhang et al.^31^ that was originally described for craniomaxillofacial applications in two-class bone segmentation and tooth and bone landmark detection. Since this approach is closely related to ours, we reimplemented the JSDNet architecture from scratch as closely as possible and applied it to the described temporal bone analysis tasks to provide an explicit comparison data point. We deduced unavailable information about architecture design or training protocol with a cursory hyperparameter search. The original formulation also did not contain augmentation protocols. We thus performed two training experiments, using the described augmentation scheme Aug$_{\alpha }$ for one instance and no augmentation for the other instance. A detailed description of the protocol for the JSDNet implementation is provided in the supplementary materials Sect. S.8.

### Training

We implemented the described modular and dual headed deep neural network architecture in PyTorch^48^. A three-fold cross validation strategy was used with dataset A to train and validate the networks in all experiments. For each fold, we randomly selected $N = 38$ datasets for training and $N = 5$ datasets for validation. The dual-head 3D-U-Net architecture was trained for a fixed pre-set of $7.5\times 10^{4}$ iterations starting from a fully random initialization. The initialization was performed using a uniform distribution. Gradient accumulation was performed over 10 iterations. For all experiments, trapezoidal learning rate scheduling with warmup, plateau and annealing phases was used. One iteration was defined as the forward pass of a batch of volume chunks, the loss computation and the computation of the corresponding gradients with respect to the network parameters. Every 750 iterations, the current model performance was measured by computing the evaluation metrics IoU and DSC for the segmentation head output, MSE and mean absolute error (MAE) for the heatmap head output as well as the validation loss, using the $N = 5{}$ validation datasets. The model parameters of both the validation iteration yielding the optimal evaluation criteria values and the last iteration were saved as the result for every training run in the employed three-fold cross validation strategy. The model parameters with optimal evaluation criteria values were used for the subsequent performance quantification with the validation and test dataset catalogue. For the ablation studies we defined a basal training configuration similar to the *BaseUNet* architecture configuration outlined above. This encompassed the AdamW optimizer with weight decay parameter $\lambda = 0.025$ and a fixed heatmap schedule. If we refer to *BaseUNet*, then architecture and training strictly adhered to the specified parameters. Again, mono-parametrically introduced variations were employed to glean the respective performance impact. For the optimizer, we tested against stochastic gradient descent (SGD). For the AdamW settings, we tested against different weight decay parameters $\lambda$ and running average coefficients $\varvec{\beta }$. For the heatmap schedule, we tested against three different amplitude and spread sequences at different iteration milestones during the training process. For gradient accumulation, we tested against a variable number of accumulation iterations with $N_{\textrm{accum}}\in \{1,5,10,15,20\}$

### Performance evaluation

We used Dataset B ($N = 9{}$ instances) as the primary test of the model performance after the conclusion of the training phase. Since B is a separate hold-out set for all experiments, it also shows the generalization skill of the model towards data sourced from clinical practice. The processed open-source datasets W, O and G were further used to determine the model performance for data from differing CT imaging setups. All testing results were produced with three-fold cross validation, yielding the mean and standard deviation of the performance over the folds.

The prediction of the joint segmentation and detection algorithm was evaluated by computing a variety of evaluation criteria^49^. We employed the spatial overlap based performance metrics Dice-Sørensen^50^ similarity score (DSC, primary metric) and Intersection-over-Union (IoU, alternatively Jaccard index)^51^ and the volumetric similarity score (VS)^52^ for the evaluation of segmentation predictions. In addition to the overlap-based metrics, we utilized the Hausdorff distance (HD) as a contour- and spatial distance based evaluation criterion to take the spatial structure and boundary delineation of the segmentation into account. In addition to the default Hausdorff distance that is susceptible to outliers, we calculated the average Hausdorff distance $\langle \textrm{HD}\rangle$, obtained from HD by supplanting the maximum operation with an average over all voxels^53^. For the ablation experiments, HD and $\langle \textrm{HD}\rangle$ was not computed due to the substantial computational complexity. The evaluation of the localization task was initialized by computing the predicted landmark coordinates through the application of the argmax operator to the output heatmaps, yielding the voxel position vector of the heatmap mode. Then, the per-landmark Euclidean distance between the ground truth voxel coordinate vector and the argmax coordinate vector was computed. Since all volumes were resampled to an isotropic voxel edge length of 99 ${\upmu }$m prior to insertion into the pipeline, we report the unit-bearing parameters Euclidean distance and Hausdorff distance hereafter in voxel units. Mathematical definitions of the metrics as used in this work are provided in the supplementary materials Sect. S.6.

## Results

**Figure 3:**
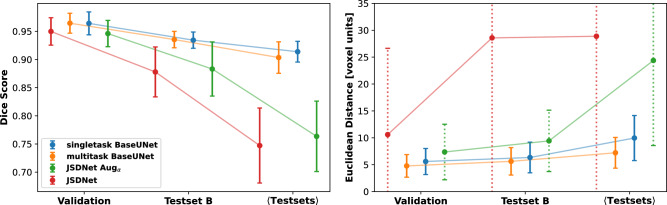
Comparison of segmentation (left) and average localization performance (right) for the basal UNet configuration trained for the respective single task (blue) and multitask setting (orange). Two manifestations of the JSDNet architecture by Zhang et al.^31^ are depicted: one trained with the default augmentation scheme Aug$_\alpha$ and one without augmentation (original formulation). Error bars show the standard deviation computed with the three-fold CV strategy.

The U-Net was trained using the training protocol outlined above from random initialization until the preset number of iterations was completed. The training loss generally exhibited stable reduction to a low value, while the validation performance reached a high and consistent plateau value (see supplementary materials Sect. S.3 for more details). These observations indicated a benign convergence behaviour of the algorithm and confirmed the successful avoidance of overfitting. We first turn to the comparison of the single-task version of the *BaseUNet* architecture with its multi-task counterpart and our reimplementation of the JSDNet by Zhang et al.^31^. The results for the overall segmentation and localization performance are shown in Fig. 3.

Segmentation wise, all models displayed excellent performance on the validation dataset with a Dice score of 0.95 for both JSDNets and 0.96 for single- and multi-task BaseUNet. On test dataset B, both BaseUNet variants exhibited similar segmentation performance with a Dice score of 0.93. The JSDNet variants suffered from a steeper performance drop-off with the augmentation-free trained variant offering slightly worse segmentation scores at 0.88. For the average performance on all hold-out datasets, the Dice score 0.90 for multi-task BaseUNet slightly differed negatively from single-task with 0.91. JSDNet again showed a larger Dice score drop with 0.76 and 0.75 for augmented and unaugmented training, respectively. For the localization task, an Euclidean distance deviation gap between single-task (5.6 voxel units) and multi-task (4.8 voxel units) was visible already on the validation data. The superior performance of the multi-task solution continued for test dataset B (5.6 voxel units) and test dataset average (7.2 voxel units). The loss of localization precision was larger for the single-task BaseUNet with 6.3 voxel units and 10.0 voxel units. JSDNet trained with augmentation had comparably larger localization deviations with 7.3, 9.4 and 24.0 voxel units for validation, dataset B and test dataset average respectively. The JSDNet instance that was fitted without training augmentations displayed even bigger landmark localization deviations with 10.6, 28.6 and 28.9 voxel units for the dataset triplet.

The next results can be gleaned from the ablation studies. For this, we conducted and evaluated additional 43 experiments with three-fold CV, where mono-parametric variations of the foundational BaseUNet architecture or training protocol were evaluated. Performance summary plots of all ablation experiments are provided in the supplementary materials Sect. S.1. Generally, training augmentation protocol and UNet network depth proved to be the largest modifier of generalization performance. Not augmenting the training data had no effect on segmentation performance and a small detrimental effect on localization ability for validation data, but was crucial for generalizing well to the test datasets. For example, the segmentation Dice score dropped by about 0.05 between best-performing augmentation protocol Aug$_\beta$ and no augmentation protocol. Similarly, the mean localization deviation increased by approximately 9 voxel units between the protocols. Furthermore, attention gating applied to both heads, heatmap scheduling and deep supervision proved to be beneficial to performance. In the realm of activation functions, Mish, GELU and SiLU exhibited stronger performance carryover to the full test set average than the traditional rectifier variants ReLU, PReLU and leaky ReLU. Loss wise, joint cross-entropy-Dice-loss exhibited the largest segmentation performance drop-off from validation to test set average but comparatively proved optimal for localization performance. For gradient accumulation, $N_{\textrm{iter}}=15$ iterations proved to be optimal in terms of average test set performance. The tested instances of SGD could not achieve competitive overall performance within the gradient update step budget and tested learning rate settings in comparison to adaptive optimizers. Notably, in comparison landmark localization was affected more negatively by the SGD optimizer than segmentation performance. For weight regularization and running average parameter settings of the AdamW optimizer, we observed a conflicting result, where parameter combinations optimal for one task were non-optimal for the other. The effect, however, was small. Normalization layer wise, instance norm exhibited better segmentation performance than batch norm for the validation and test dataset B. Batch normalization then yielded better mean test-set average performance with enlarged standard deviation. For localization performance, the situation was inverted and batch normalization performance was superseded by instance normalization performance on the test set average.

**Figure 4:**
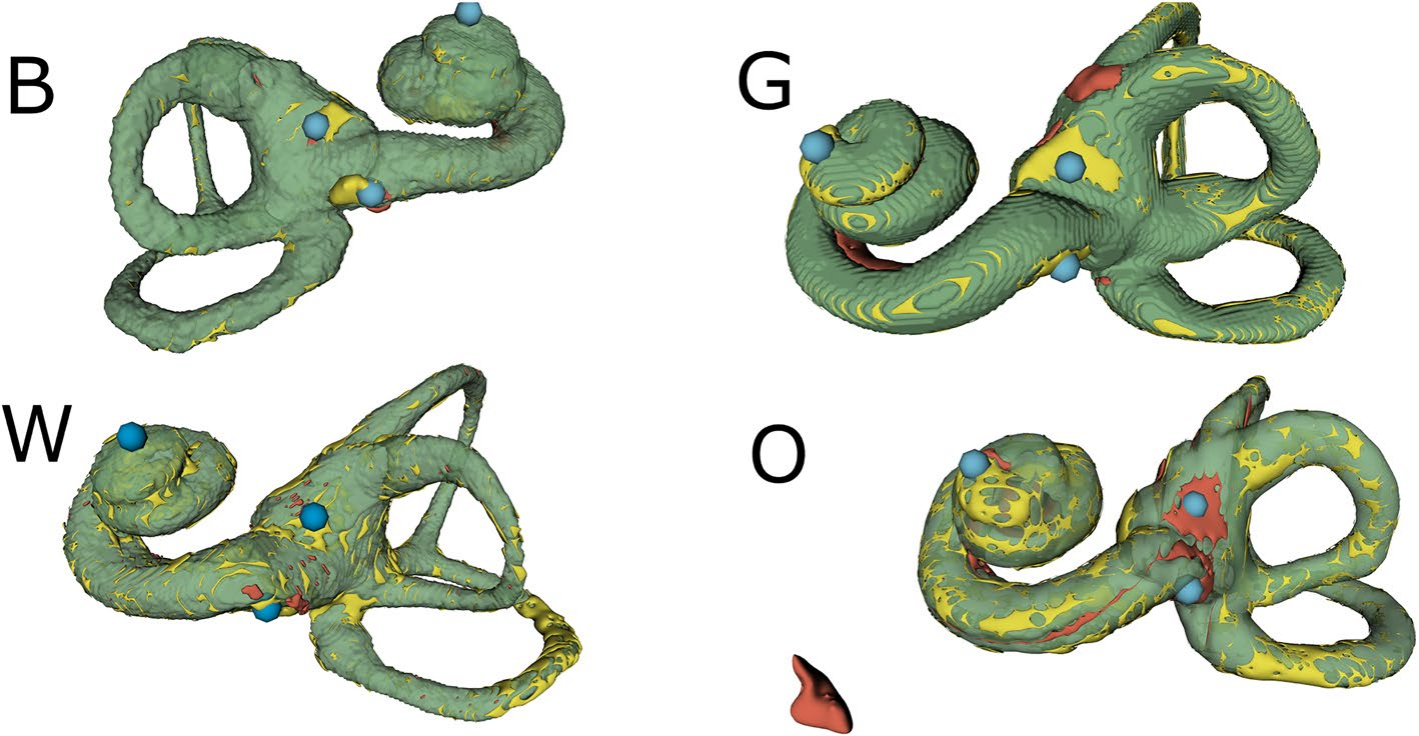
3D overview of prediction results from the test datasets B (upper left), G (upper right), W (lower left) and O (lower right). The machine prediction true positive volume is depicted in green. Compared to the ground truth (i.e. the manual labels), false positive or oversegmented voxels are colored in red while undersegmented or false negative classified voxels are colored in yellow.

We then produced the final resulting *gold-status network architecture* via a manual and greedy collection of task-optimal design choices using insights generated from the ablation study. The resulting architecture encompassed global attention gating, instance normalization, Mish nonlinearity, augmentation protocol Aug$_\beta$, heatmap scheduling protocol $\gamma$, deep supervision and gradient accumulation over 15 iterations. For this experiment, overall quantitative evaluation results for the segmentation criteria DSC, IoU, VS and HD are jointly reported in Table 3 for the validation, hold-out clinical test and open-source datasets. The corresponding landmark coordinate regression evaluation results are stated in Table 4. An excellent segmentation performance on the validation ($\textrm{DSC}= {0.97}(2)$) and test ($\textrm{DSC}= {0.94}(1)$) dataset was observed. As indicated by the quantitative scores, the automated segmentation revealed to be anatomically realistic with small to none erroneous volumes apart from the main inner ear structure. For the open source dataset W ($\textrm{DSC}= {0.94}(2)$) a segmentation Dice score comparable to the test dataset B was observed. Dataset O exhibited reduced segmentation performance with $\textrm{DSC}= {0.89}(3)$. Segmentation map predictions for the final open-source Dataset G also had slightly reduced overlap, resulting in an overall Dice score of $\textrm{DSC}= {0.91}(2)$ for this microCT dataset. The landmark localization performance generally followed suit, with an average localization deviation for the framework of 3.3 voxel units and 5.2 voxel units for validation and test set B. Similar to the segmentation result, the localization deviation for the instances of dataset W was the lowest of the open-source test datasets with an average deviation of 5.0 voxel units. The localization performance difference for predictions of the remaining test datasets O and G in comparison to the in-house validation datasets was comparatively smaller, as the automated network was able to locate the landmarks with an average deviation of 5.4 voxel units for dataset O and 5.8 voxel units for dataset G (i.e. comparable to localization accuracy on the datasets B and W). A set of illustrative 3D renderings of automated machine prediction results on multiple test dataset instances is shown in Fig. 4. The images show the true positive prediction volume itself (green) as well as the false negative (yellow) and false positive (red) voxel classifications. We selected the actual instances such that the instance Dice overlap score of the shown instance prediction matches the global dataset average score provided in Table 3, yielding representative dataset-wise visualizations. The ground truth positions landmarks helicotrema, oval and round window are marked by blue spheres. The spheres possess a radius of 10 voxel units, providing a visual cue and standard measure for the reported voxel unit deviation values. As shown in Table 4, the average localization deviation for all datasets is well below the shown blue deviation sphere.

**Table 3:** Evaluation result of the gold-status network for the segmentation subtask for the different datasets.

	DSC			IoU			VS			$\textrm{HD}$			$\langle \textrm{HD}\rangle$		
ID	Mean	SD	MD	Mean	SD	MD	Mean	SD	MD	Mean	SD	MD	Mean	SD	MD
A	0.97	0.02	0.98	0.94	0.03	0.95	1.00	0.03	1.00	6.4	3.9	5.4	0.06	0.04	0.06
B	0.94	0.01	0.95	0.89	0.02	0.90	0.95	0.02	0.96	9.7	19	9.1	0.14	0.10	0.11
W	0.94	0.02	0.94	0.89	0.03	0.89	0.95	0.03	0.95	12	14	7	0.15	0.13	0.13
O	0.89	0.03	0.88	0.80	0.04	0.79	0.96	0.04	0.95	56	40	67	0.68	0.84	0.43
G	0.91	0.02	0.90	0.83	0.03	0.82	0.97	0.02	0.97	8.8	2.8	8.3	0.15	0.04	0.15

Hausdorff distance values are stated in voxel units. SD denotes standard deviation, MD denotes median.

**Table 4:** Evaluation result for the landmark localization subtask for the different datasets.

	Average			Helicotrema			Oval window			Round window		
ID	Mean	SD	MD	Mean	SD	MD	Mean	SD	MD	Mean	SD	MD
A	3.3	1.5	3.1	4.8	2.1	4.7	2.9	1.5	3.0	2.3	1.0	1.7
B	5.2	2.8	4.5	6.8	4.8	4.7	4.3	1.4	4.6	4.5	2.2	4.1
W	5.0	1.9	4.9	7.5	2.4	7.3	3.3	1.5	3.3	4.1	1.7	4.1
O	5.4	2.5	5.2	7.0	3.2	6.0	4.6	1.8	5.1	4.6	2.4	4.6
G	5.8	2.5	5.5	7.4	2.5	7.3	5.5	3.1	5.1	4.3	2.0	4.0

The Euclidean distance was computed between the prediction and the manually labeled data and is given in voxel units. SD denotes standard deviation, MD denotes median.

For dataset O, the selected instance shown in Fig. 4 visibly contained a separate false-positive volume. The performance of the framework in that regard can be gauged from the Hausdorff metric values provided in the latter part of Table 3. The basal Hausdorff distance of 6.4 voxel units and 9.7 voxel units for the validation and test set B show the dataset-aggregated worst case for misclassified volumes. For the open-source dataset O, this observed HD value was more than five-fold enlarged compared to test dataset B. Its averaged companion value increased similarly about five-fold.

Apart from the qualitative considerations, the efficiency benefits of the proposed solution can be stated. In this study, the time to manually and accurately label the relevant anatomical structures and landmarks in a new dataset instance was approximately 600 s using the described threshold-based masking and manual painting method. For the basic, GPU-accelerated PyTorch implementation, the average prediction time amounted to 50 s for a typical input volume with a TTA strategy of ten augmenting transformations and 25 voxels overlap per sub-volume chunk. This time included loading the dataset instance from disk.

**Figure 5:**
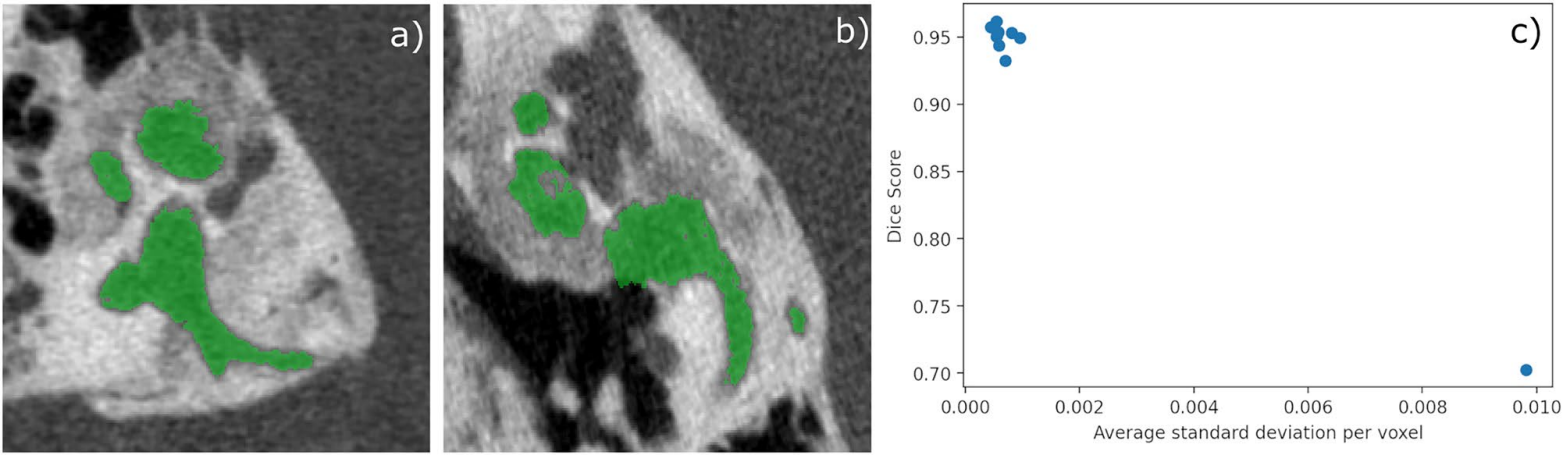
Two differing slice views of raw data overlaid with ground truth annotation in (**a**) and (**b**) of the clinical dataset instance with otosclerotic lesions. The corresponding 2D plot of the average prediction standard deviation per voxel (*x* coordinate) against the prediction Dice score (*y* coordinate) in panel c) shows the distinct clustering of the non-pathological datset instances (upper left corner) with high segmentation performance and low standard deviation apart from the otosclerotic instance (lower right corner) with low segmentation performance and higher standard deviation. The TTA augmentation and standard deviation computation scheme might be leveraged to perform a preliminary uncertainty quantification.

In addition to the evaluation of the 9 clinically regular instances of dataset B, the single otosclerotic dataset instance was also processed with the described pipeline and algorithm. A significantly lowered segmentation performance as well as landmark localization performance compared to the regular instances of dataset B was observed. The ground truth segmentation map is depicted in Fig. 5a and b for two exemplary slice images. With the TTA protocol, we additionally computed the aggregation and standard deviation of the prediction supersamples. The comparison of the mean of the volume-average standard deviation per voxel for the 9 instances and the corresponding parameter of the singular otosclerotic instance showed an approximately 15-fold increase from ${6.4}(15)\times 10^{-4}$ to ${9.81}\times 10^{-3}$.

## Discussion

Motivated by the resource constraints and drawbacks of manual analysis of volumetric radiological temporal bone images, an automated pipeline-based framework to jointly produce inner ear segmentation maps and localize three important anatomic landmarks without the requirement of user interaction was developed. After loading the a dataset instance, no further user interaction was necessary for the production of a scan analysis that consisted of the prediction of the inner ear anatomy (union of cochlea, vestibulum and semicircular canals) and the coordinate-wise localization of the three important anatomical landmarks (helicotrema, oval and round window).

The primary objective of machine learning is to optimize a function with limited training data that generalizes well to genuinely new and unseen data instances.

The described results provide an interesting evaluation due to the fact of being trained on frozen cadaveric specimen only, while being tested on scans from clinical practice (see Table 2) obtained with similar scanning parameters.

The generalization ability to practical, real world clinical settings could be demonstrated through the markedly high DSC scores and good localization accuracy on the held-out test data of B.

When compared with the catalogue of related works presented in Table 1, the specified framework was able to achieve a superior segmentation performance Dice score of 0.97 and 0.94 for the core validation and test datasets respectively. This performance gain also translated into the total test dataset average, where the Dice value 0.92 outperformed the inner ear segmentation scores of all related works. For example, Neves et al. reported a Dice score of 0.91(3) for their AH-Net^15^ while Hussain et al. accomplished a score of 0.900(70) with AutoCasUNet^17^. The proposed framework was able to predict segmentation maps with a Dice score of 0.91(2) for the dataset G in a hold-out test setting, enabling direct commensurability to Hussain et al., who also employed dataset G as core training and validation data.

We note however that for performance results to be commensurable in all aspects, a single authoritative benchmark dataset like the medical segmentation decathlon dataset^54^ should be created for inner ear and temporal bone segmentation. This strategy would help to disentangle performance influences that are not genuinely attributable to pipeline and algorithm. Possible candidates therefore are e.g. the voxel size, labeling quality and basal CT imaging setup.

For the localization task, the method was able to provide highly accurate localization of all three anatomical landmarks (see Table 4 and 10 voxel unit deviation spheres in Fig. 4) that were below the general feature size of the membraneous structure in the case of the oval and round window. Interestingly, for all dataset-wise evaluations, the localization distance error of the helicotrema was generally larger compared to the oval and round window distance error. We judged this as a surprising result, since the helicotrema is expected to be generally located on a bone-cavern interface with high radiodensity gradient that should be well captured by the segmentation. We surmise that the ground truth labels for the differing instances are subject to considerable variations due to the difficulty of determining the true most extremal point of the cochlea with respect to the modiolar axis.

Apart from numerical quantification with congruence scores, we examined the spatial error distribution to provide insights about the ability of the methodology to produce segmentations that are clinically deployable, e.g. for the envisioned 3D visualization of inner ear anatomy for intervention planning and reviewing. For the core dataset B from clinical origins, the model mostly exhibited a benign error behaviour, meaning that voxel misclassifications were generally restricted to the transitions from osseous structures to cavities (see false positive and false negative error voxels in Fig. 4), i.e. thin interface volumes with a large radiodensity gradient. In addition to transition surfaces, inherently difficult to segment regions like ambiguous soft-tissue membranes (e.g. oval, round window) and nerve tissue (e.g. cochlear nerve) were another focal region of increased misclassifications.

Our design choice to not employ postprocessing algorithms like largest region extraction or probabilistic active shape modeling opens up the possibility of dissociated erroneous false positive misclassifications. As an advantage, this design choice allows to gauge the raw performance of the learned algorithm since all metrics are computed on direct model outputs. In an actual clinical application setting, misclassified regions apart from the main structure would require manual cleaning. For this real-world use, postprocessing like registration to shape models or largest region extraction will be beneficial from a pure segmentation quality standpoint. This observation of misclassification being located close to the ground truth is underlined by the small average Hausdorff distance (see Table 3) values, due to the fact that the metric quantifies the average distance-based error of the machine prediction, in contrast to the volumetric and congruence error quantified by Dice, Intersection-over-Union and volumetric similarity. The maximum Hausdorff distance as an outlier-sensitive metric provides further insights, indicating the existence of some separate misclassification compartments. This is more pronounced for the open-source datasets, especially for dataset O. We assume that the contrast alterations induced by the various preservations methods of the open source datasets, with the procedure of O being the most invasive one with drilling and multiple immersions in resins and solutions, effect a data domain shift that is not fully corrected for even after specialized processing. Summarizingly, we anticipate that the described benign error behaviour of the automated framework permits a manageable integration of fully automated cochlear duct length measurement into the developed segmentation and localization framework.

The inclusion of test-time augmentation proved to be beneficial in two differing aspects for in-house datasets and external open-source datasets respectively. Namely, the performance improvement for the open-source datasets was accompanied by the complementary use of TTA-produced prediction supersamples in the in-house datasets to create information about uncertainty. The open-source datasets profit off TTA by improved segmentation and localization performance for the voxel-wise average prediction map. With TTA, the network receives multiple rotational point of views for the data that was originally not oriented according to standard operating clinical procedure. On the other hand, TTA could be employed to compute prediction uncertainty maps from the multiple TTA prediction supersamples. These standard deviation maps (exemplary depiction in supplementary materials S.2) may be interpreted as a tentative prediction uncertainty estimation and can thusly provide users with additional spatial confidence maps. The observable congruence of the true positive and true negative misclassification maps and the prediction supersamples standard deviation map encourages this approach of TTA-based uncertainty estimation. This can facilitate the applicability of data-driven automated models by helping to identify overconfident incorrect predictions^55^. Thus, the inclusion of TTA proved to be a valuable element of proposed framework for possible applications in mission-critical scenarios like preoperative planning for cochlear implantation.

The dataset instance of B which contained otosclerotic lesions (see contrast alterations in the label surroundings in panel a) and b) in Fig. 5) was processed with markedly lower segmentation and localization performance by the automated framework. The larger misclassified volumes were caused by the contrast and bone texture alterations present in the otic capsule that is being pathologically remodeled during otosclerosis. Thusly, this dataset instance presents itself as an interesting, anatomy-wise out-of-distribution performance gauge. With a default, forward-styled reasoning we conclude from the diminished performance that the devised system trained with the described dataset is less applicable to pathological input data.

However, we noted that the global volume-mean standard deviation per voxel is substantially larger than the corresponding value for all non-pathological dataset instances. In Fig. 5c), where the Dice score is plotted against the prediction samples standard deviation, this clear separation is evident. We hence speculate in a line of inverted reasoning that TTA in conjunction with the computation of prediction standard deviation may have a possible use case as out-of-distribution and subsequently pathology detection mechanism. The voxel-wise mean standard deviation of predictions can be computed without any ground truth information and raised values for this parameter may serve as a signal for pathologies. Further investigations with more dataset instances featuring otosclerotic lesions could elucidate this tentative connection.

The 10-fold reduction in analysis generation time in comparison to manual labeling is an impressive demonstration of the efficiency gains of the automated pipeline. Using task automation, large scale and multi-centered clinical studies are within reach that may have been unattainable with the earlier lengthy manual methodology. Rapid generation of patient-specific anatomic analysis also facilitates the integration into routine pre- and intra-operative planning for cochlear implant surgeons. The prediction time of the pipeline can potentially be minimized even further by providing a more optimized implementation. For example, a parallelization strategy of the preprocessing TTA augmentation computation could prove feasible. The basal model inference time could be reduced by network pruning and sparsification of the U-Network or by applying weight quantization.

Despite the consideration of all presented performance metrics and efficiency gains, clinical applicability still requires a much more in-depth validation of this work, where we presented a well-performing formulation for joint segmentation and landmark localization. Segmentation wise, Neves et al.^15^ presented a blinded review with seven expert participants, where automated segmentations with a Dice overlap score of 0.91 were rated highly for accuracy. Since the described pipeline surpasses this segmentation performance, the results are encouraging with respect to real world applications. Concerning the localization performance and the potential future use for automated cochlear duct length (CDL) measurements, we note that for clinical applications the translation of the error propagation from helicotrema and round window localization into the final CDL is relevant. The proposed framework was able to locate helicotrema and round window in clinical data with a mean deviation of 6.4 and 4.5 voxel units. Using a cursory worst-case error analysis with physical units (via the voxel edge length of 99 $\upmu$m), the worst-case error for CDL measurements initialized by both landmark positions amounts to a value of 1.08 mm. For example, Taeger et al.^56^ showed that CDL measurement deviations of this magnitude are not clinically relevant with respect to the tonotopic frequency layout of the cochlea.

### Limitations

Due to the pervasive large need for data of deep learning methods, one impediment of the presented work is the relatively small number of dataset instances utilized to train and test the learned model.

Further testing with (clinical) CT datasets that do not suffer from the inner ear contrast alterations and rotational differences observed in the preserved specimen open-source dataset and thus requiring enhanced processing is desirable.

The bottleneck for the labeling of more in-house instances was the time-intensive process of manual segmentation. The high segmentation and localization performance of the framework may provide potential to *bootstrap* a larger collective of datasets via prediction of newly recorded volumes that get quickly reviewed, refined and reintroduced into the training dataset. In our evaluation, we focused on healthy subjects and explicitly treated a pathological dataset instance as out-of-distribution. Special care should be given to the collection of more pathologically altered datasets (e.g. otosclerosis and fibrosis) to better understand the out-of-distribution performance of the model on these dataset instances. The data sourcing for these cases is often even more difficult than for non-pathological cases and may necessitate further specialized data augmentation or transfer learning strategies.

Another considerable limitation is the absence of fully authoritative ground truth data concerning the segmentation maps and the landmark positions. The ground truth data of datasets A and B was manually generated by a researcher trained and supervised by a qualified clinical researcher and otological surgeon. Subsequently, intra-observer variability may exist in datasets A and B that effect a systematic labeling difference with the external open source dataset catalogue. Incorrect or inconsistent segmentation masks that are considered the absolute ground truth could thusly lead to a low DSC evaluation for the automated prediction. In this possible scenario, the lower DSC would paradoxically indicate a better automated prediction in comparison to the manual annotation. A viable remedy would be a general input data improvement strategy using a multitude of anatomical experts that jointly segment all dataset instances, from which a gold standard and consensus ground truth could be distilled. Such an endeavour could potentially culminate in the development of the earlier mentioned authoritative *benchmark dataset* for temporal bone segmentation and landmark detection, improving commensurability and reproducibility of the various automated methodologies.

### Prospects

Directly addressing the mentioned data limitation, a very worthwhile further line of work could be a scope-maximized evaluation study using more temporal bone CT data. This would ideally entail datasets exhibiting realistic inner ear fluid space contrast and volume orientation according to clinical standard operating procedure. Using the clinical CT data generated by the related works summarized in Table 1, this would be an interesting and attainable evaluation study. Looking beyond the described framework and immediate performance considerations, we identify integrated analysis of other, possibly more complex anatomical parameters like cochlear duct length measurements, cochlear modiolar axis detection and registration^57^ within a unified fully automated pipeline as a work target. The inclusion of transfer learning and study of learning from multi-modal imaging data including MRI strikes us as another salient line of research. Our future investigations and enhancements to the methodology will focus on these beneficial objectives.

## Conclusion

The presented automated 3D U-Net framework and data processing pipeline provided highly accurate segmentations of the inner ear structure combined with good localization of the anatomical landmarks helicotrema and oval and round window via heatmap regression.

The practical applicability was demonstrated via the evaluation on representative flat-panel volume CT data with secondary reconstruction sourced from actual clinical practice. Further robust generalization skill was shown on a three-element catalogue of processed open source datasets.

The designed model incorporated state-of-the-art deep learning techniques and was implemented with a modular architecture using open source software libraries. Conducted mono-parametric ablation studies illuminated the performance impact of architectural design choices and training protocol hyperparameters and allowed the inference of task-optimal parameters. The bi-headed U-Net based core architecture was end-to-end trainable without postprocessing, enabling insights into the raw performance of deep learning based segmentation and landmark localization models. Ablation studies of the bi-headed solution against single-task formulations yielded a clear multi-task performance benefit for landmark localization. For segmentation performance, equal automated model skill with a small loss in generalization skill to open-source data was visible. In comparison to related works, we were able to show a superior joint segmentation and localization ability. With its encouraging evaluation results, the automated methodology yielded good generalization ability such that the proposed, proof-of-concept framework may possess—with significant improvements and further validation—the potential to be utilized in both clinical practice and research. Immediate, tangible applications of the segmentation and localization results comprise e.g. preoperative planning, intraoperative visualization and large scale exploratory clinical research.

### Supplementary Information

Supplementary Information 1.

## Data Availability

The in-house datasets and labels of dataset A generated and processed for this study are publicly available as a dataset contribution on Zenodo under the DOI:10.5281/zenodo.8277159. The open source datasets W^36^, O^37^ and G^18^ are publicly accessible under the provided references.
