# Supplementary material for: Towards fully automated inner ear analysis with deep-learning-based joint segmentation and landmark detection framework
Source: Sci Rep. 2023 Nov 4;13:19057. doi: 10.1038/s41598-023-45466-9 (PMC10625555; doi:10.1038/s41598-023-45466-9)
Supplement: Supplementary file 1 — Supplementary Information 1. [file 41598_2023_45466_MOESM1_ESM.pdf]

Supplementary Material

Towards fully automated inner ear analysis with deep-learning-based joint segmentation and landmark detection framework

Jannik Stebani[1,2,5] Martin Blaimer[1] Simon Zabler[1,6] Tilmann Neun[3]
Daniël M. Pelt[4] Kristen Rak[5]

[1]Fraunhofer Institute for Integrated Circuits IIS, Magnetic Resonance and X-Ray Imaging Department, Würzburg, Germany
[2]Universität Würzburg, Experimentelle Physik V, Würzburg, Germany
[3]Universitätsklinikum Würzburg, Institute for Diagnostic and Interventional Neuroradiology, Würzburg, Germany
[4]Universiteit Leiden, Leiden Institute of Advanced Computer Science (LIACS), Leiden, The Netherlands

[5]Universitätsklinikum Würzburg, Department of Oto-Rhino-Laryngology, Plastic, Aesthetic and Reconstructive Head and Neck Surgery and the Comprehensive Hearing Center, Würzburg, Germany
[6]Deggendorf Institute of Technology, Faculty of Computer Science, Deggendorf, Germany

Address for correspondence: `jannik.stebani@iis.fraunhofer.de`

Contents

S.1 Ablation Experiment Results **1**

S.2 Additional Prediction Visualizations **7**

S.3 Training Procedure Details **7**

S.4 Data Processing and Augmenting Transformations **8**

S.5 Descriptive Dataset Statistics **13**

S.6 Performance Metrics Description **17**

S.7 Network Architecture Definitions **18**

S.8 Implementation and Training of JSDNet **19**

S.1 Ablation Experiment Results

To elucidate the effect of the activation function, we performed experiments with ReLU, PReLU, LeakyReLU, GELU and Mish. The performance comparison is shown in Sup. Fig. 1. We further tested the effect of training data augmentation. Two different augmentation schemes, Aug_α and Aug_β were tested against no augmentation at all. The augmentation protocols are described at the end of this section. The basal definitions of the augmentations are given in section S.4. The result of this ablation

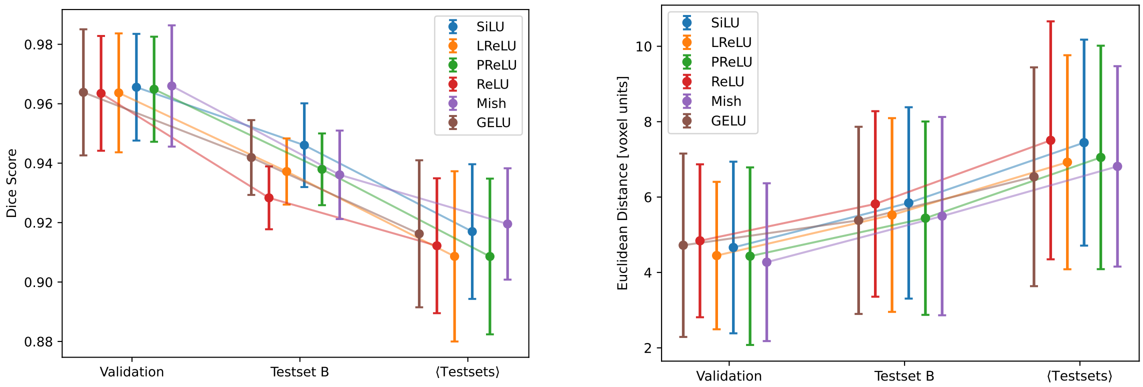

Supplementary Figure 1: Segmentation (left) and localization (right) performance comparison for the activation function ablation study. Starting from the base architecture, the activation function was mono-parametrically changed. For every configuration, the mean performance and standard deviation over the three-fold CV experiment is provided.

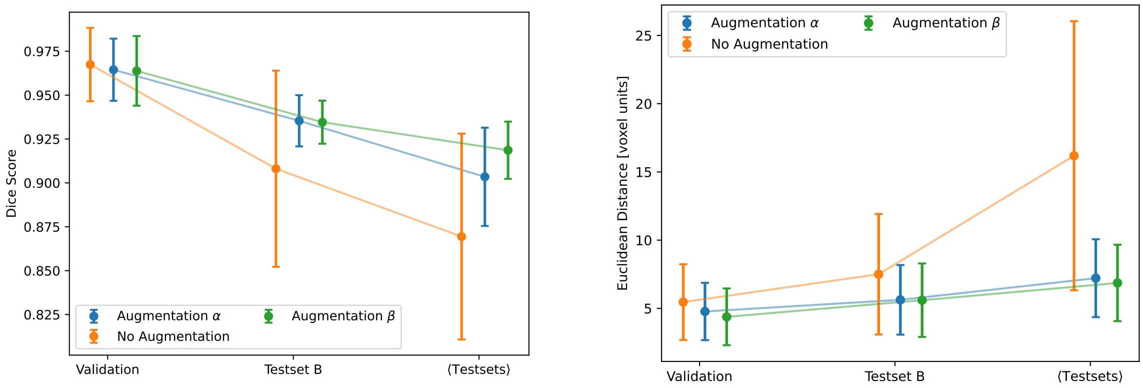

Supplementary Figure 2: Segmentation (left) and localization (right) performance comparison for the ablation study concerning training data augmentation. Two different augmentation schemes (Aug_α, Aug_β) and no augmentation are compared. For every configuration, the mean performance and standard deviation over the three-fold CV experiment is provided.

experiment is shown in Sup. Fig. 2. For the depth ablation studies, we decreased the UNet depth or channel dimension in the lowest encoder block in comparison to the basal architecture with its five-level deep feature map cascade. We experimented with the removal of the two lowest encoder elements and the channel count decrease by 64. This yielded the following feature map specifications:

- base: $[32, 64, 128, 256, 320]$
- channel reduced: $[32, 64, 128, 256, 256]$
- depth-1 : $[32, 64, 128, 256]$
- depth-2 : $[32, 64, 128]$

We show the corresponding comparison plot in Sup. Fig. 3. The next element of the ablation study

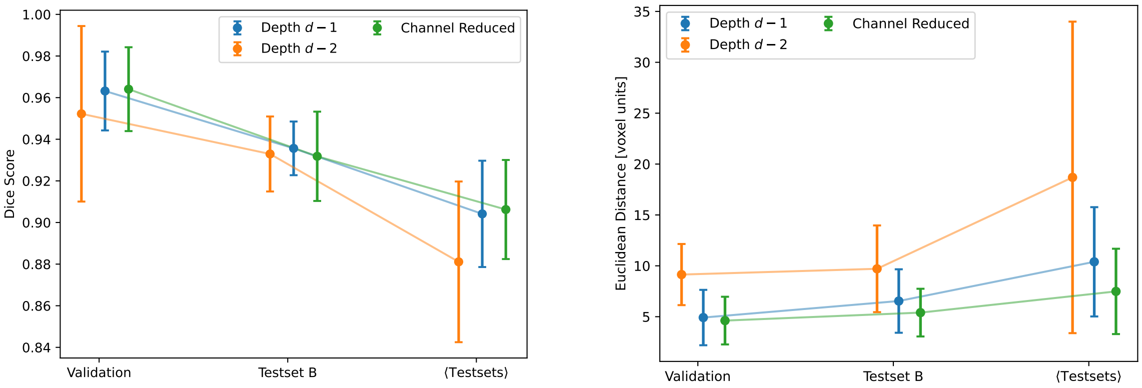

Supplementary Figure 3: Segmentation (left) and localization (right) performance comparison for the ablation study concerning network depth. We used the following configurations: channel reduced $[32, 64, 128, 256, 256]$, depth $d-1$ $[32, 64, 128, 256]$, depth $d-2$ $[32, 64, 128]$. For every configuration, the mean performance and standard deviation over the three-fold CV experiment is provided.

contained experiments with the loss function. We tested multiple variants for the segmentation-specific loss term: cross entropy with Dice loss (BCEDiceLoss), pure Dice Loss, squared Dice loss [8], Dice loss wrapped in hyperbolic cosine and logarithm [2] (LogCoshDiceLoss) and the basal configuration with disabled deep supervision. Displayed results can be found in Sup. Fig. 4. Another element of the

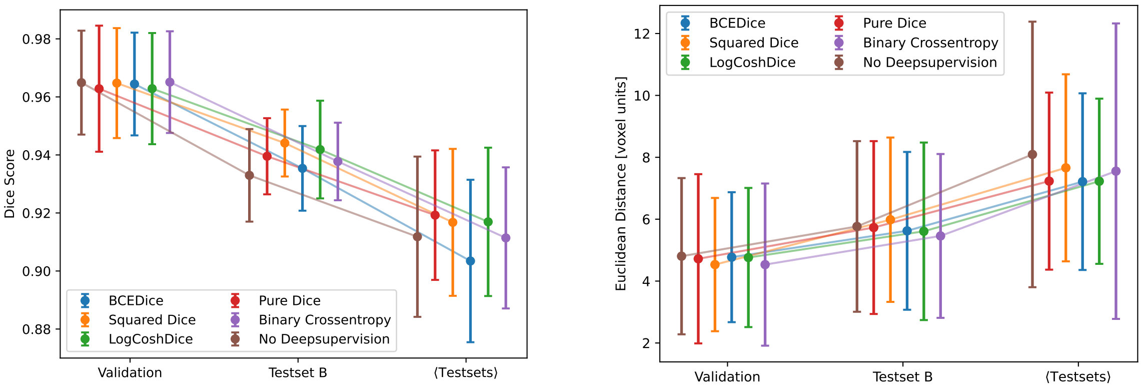

Supplementary Figure 4: Segmentation (left) and localization (right) performance comparison for the ablation study concerning the various loss functions based on cross entropy and Dice coefficient. For every configuration, the mean performance and standard deviation over the three-fold CV experiment is provided.

presented pipeline was heatmap scheduling, i.e. the adjustment of heatmap amplitude and spread during the training process at preset milestones. The results for our tests with three different schedules and no scheduling are shown in Sup. Fig. 5. The adaptive optimizer AdamW [6] has multiple parameters that modify its gradient running average calculation and weight decay regularization. We performed ablation

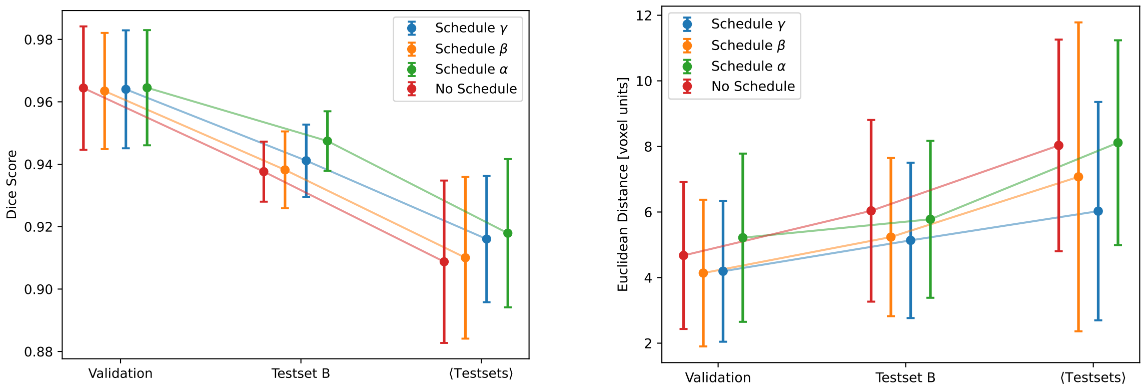

Supplementary Figure 5: Segmentation (left) and localization (right) performance comparison for the ablation study concerning heatmap scheduling. Three different augmentation schemes (α, β, γ) and no heatmap schedule are compared. For every configuration, the mean performance and standard deviation over the three-fold CV experiment is provided.

experiments with four different combinations for the weight decay parameter λ and the running average parameters $\boldsymbol{\beta}$. The results for the experiments with different AdamW configurations are shown in Sup. Fig. 6. Besides the adaptive optimizer `AdamW`, we performed experiments with plain stochastic gradient

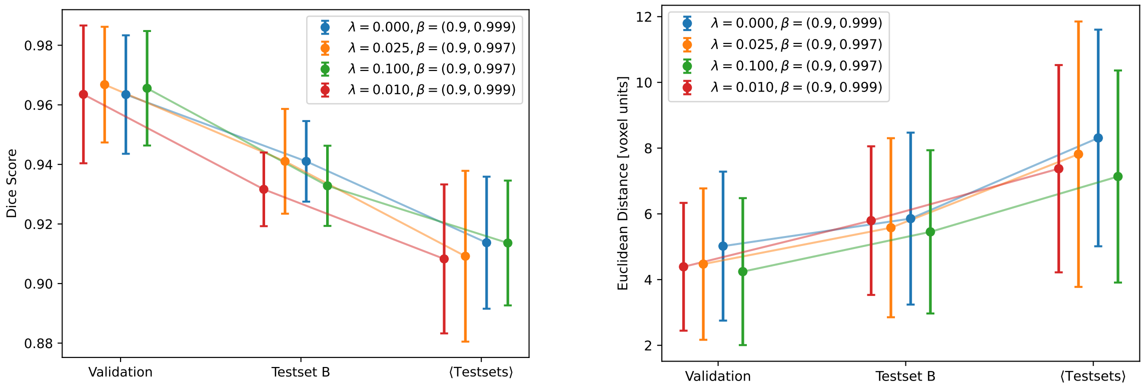

Supplementary Figure 6: Segmentation (left) and localization (right) performance comparison for the ablation study concerning different running average and weight regularization parameters settings for the adaptive optimizer AdamW. Four different combinations were tested. For every configuration, the mean performance and standard deviation over the three-fold CV experiment is provided.

descent (SGD) with three different learning rates $\eta \in \{7.5 \times 10^{-4}, 1 \times 10^{-3}, 5 \times 10^{-3}\}$. The ablation experiment results for SGD are exhibited in Sup. Fig. 7. Another building block in the architecture was attention gating [9]. For the ablation studies with this element, we slightly deviated from the otherwise mono-parametric ablation studies by testing attention gating tentatively with other variations of the BaseUNet architecture and training protocol. For attention gating alone, we tested the global application and selective application to either task-specific output head. For data series without prefix, global attention is applied. Selective application to the segmentation or localization head is signified by the "Seg" or "Loc" prefix respectively. Variations in addition to attention gating included the SGD optimizer with $\eta = 7.5 \times 10^{-4}$, batch normalization layers, PReLu and Mish activation functions and the training data augmentation scheme Aug_β. The performance comparison plot for the attention gating ablation study is given by Sup. Fig. 8. We accumulated the gradients over multiple iterations inside our training loop due to the small batchsize of two volume chunks. The accumulation iterations were another element in the ablation studies and we tested accumulation iteration counts from 1 up to 20. The results for this ablation experiment is shown in Sup. Fig. 9. The last tested element in the ablation experiments was the normalization layer type. We tested instance normalization [11] against batch normalization [1]. The results of this experiment are displayed in Sup. Fig. 10.

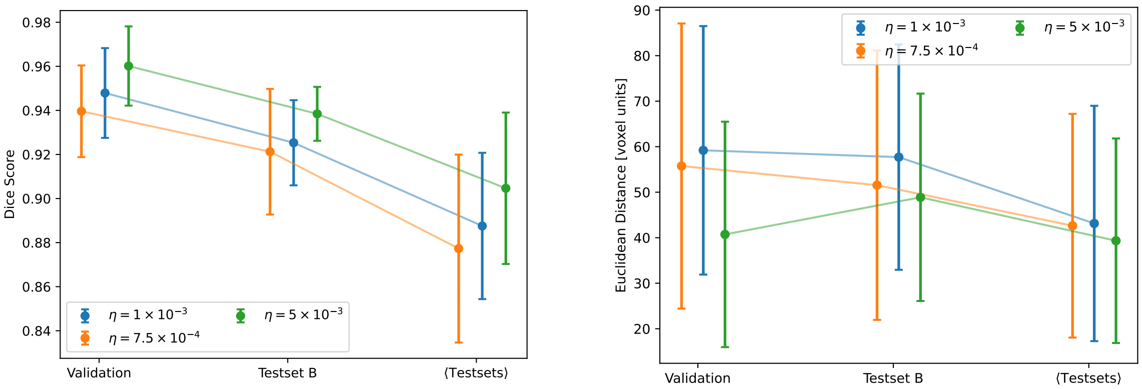

Supplementary Figure 7: Segmentation (left) and localization (right) performance comparison for the ablation study concerning plain stochastic gradient descent instead of adaptive optimizer AdamW. Three different learning rates were tested. For every configuration, the mean performance and standard deviation over the three-fold CV experiment is provided.

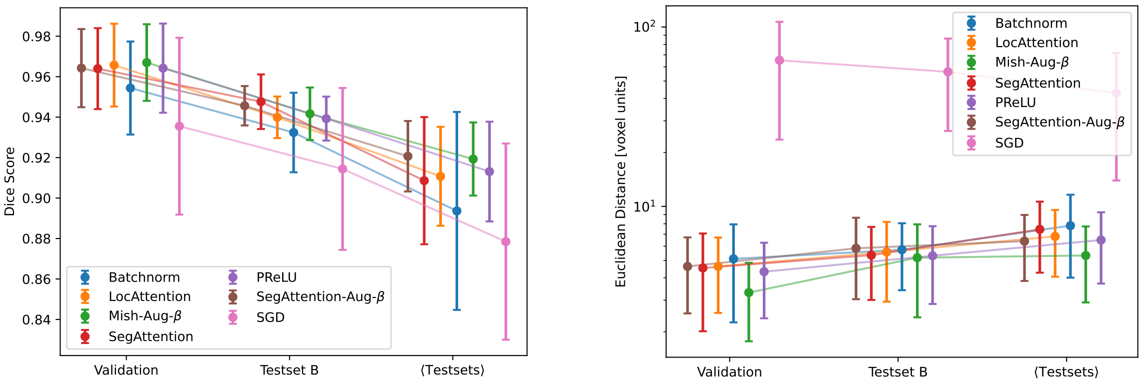

Supplementary Figure 8: Segmentation (left) and localization (right) performance comparison for the ablation study concerning attention gating. No prefix indicates attention gating applied to both heads, and "Seg" or "Loc" application to the respective task specific head. For every configuration, the mean performance and standard deviation over the three-fold CV experiment is provided.

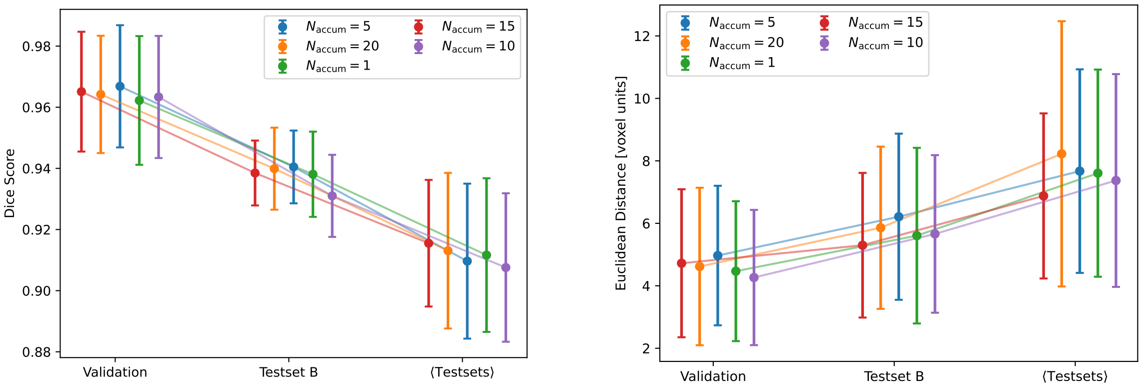

Supplementary Figure 9: Segmentation (left) and localization (right) performance comparison for the ablation study concerning gradient accumulation. For every configuration, the mean performance and standard deviation over the three-fold CV experiment is provided.

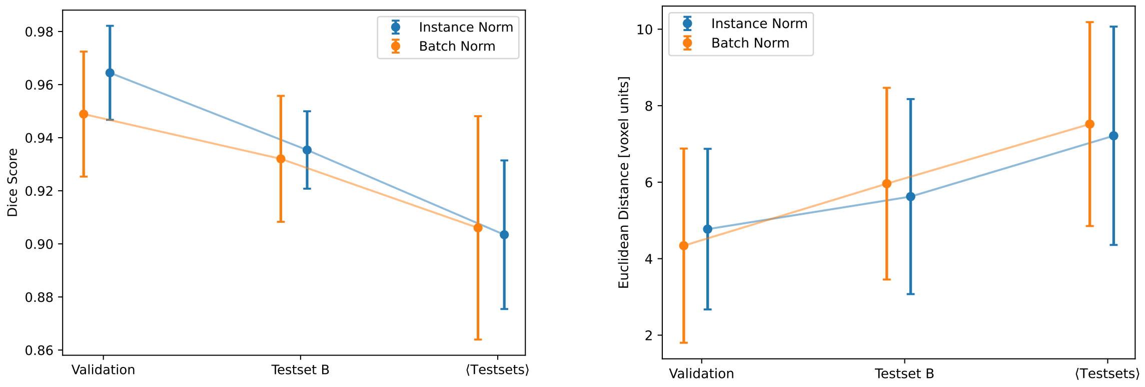

Supplementary Figure 10: Segmentation (left) and localization (right) performance comparison for the ablation study concerning the normalization layer type. For every configuration, the mean performance and standard deviation over the three-fold CV experiment is provided.

Training Augmentation Protocols For the training data augmentation, we utilized two differing protocols that were termed Aug_α and Aug_β.

- training augmentation variant Aug_α
  - random rotation of 90° or 180° or 270° about the first I axis of the voxel volume
  - Flipping about any axis $\{I, J, K\}$ with a per-axis probability of 0.5
  - free angle rotation about any axis with a maximum angle amplitude of 30°, actual value randomly sampled
  - additive Gaussian noise with a noise scale σ drawn from the interval $[0.25, 0.95]$ and an execution probability of 0.33
  - contrast transformation with a scaling factor drawn from the interval $[0.5, 2.0]$ and an execution probability of 0.25
- training augmentation variant Aug_β
  - random equiprobable selection of one of the following augmentations:
    - random rotation of 90° or 180° or 270° about the first I axis of the voxel volume
    - random rotation of 90° or 180° or 270° about the second J axis of the voxel volume
    - random rotation of 90° or 180° or 270° about the third K axis of the voxel volume
    - Flipping about any axis $\{I, J, K\}$ with a per-axis probability of 0.25
  - free angle rotation about any axis with a maximum angle amplitude of 45°, actual value randomly sampled
  - additive Gaussian noise with a noise scale σ drawn from the interval $[0.33, 1.125\}$ and an execution probability of 0.5
  - contrast transformation with a scaling factor drawn from the interval $[0.5, 2.125]$ and an execution probability of 0.5

Heatmap Scheduling For the heatmap augmentation protocol we employed three different subvariants with the indices α, β and γ. The core scheduling process adjusts the amplitude and spread parameter of the heatmaps during the training process. The adjustment was performed according to a preset epoch schedule. An epoch was defined as a full iteration trough the training dataset. The milestones were defined as fractions or percentages of the total number of epochs. Due to our fixed training iteration count of 75000, these relative milestone specifications are commensurable for all conducted experiments. The heatmaps for all landmarks were then dynamically recomputed on the attainment of the epoch milestone. The spread parameter can be interpreted as radius of the spherical heatmap in voxel units since the heatmaps were computed on the IJK voxel grid. We utilized the following three variations for the schedule:

- schedule variation α
  - amplitude parameter sequence $(2.5, 2.5, 3.5, 5.0)$
  - spread parameter sequence $(25, 20, 15, 12.5)$
  - epoch milestone sequence: $(20\%, 40\%, 60\%, 75\%)$
- schedule variation β
  - amplitude parameter sequence $(2.5, 2.5, 3.25, 3.5, 4.0)$
  - spread parameter sequence $(25, 20, 15, 12.5, 10.0)$
  - epoch milestone sequence: $(15\%, 25\%, 40\%, 50\%, 65\%)$
- schedule variation γ
  - amplitude parameter sequence $(2.5, 2.5, 3.25, 3.5, 4.0)$
  - spread parameter sequence $(22.5, 20, 15, 10, 5.0)$
  - epoch milestone sequence: $(10\%, 20\%, 30\%, 45\%, 55\%)$

S.2 Additional Prediction Visualizations

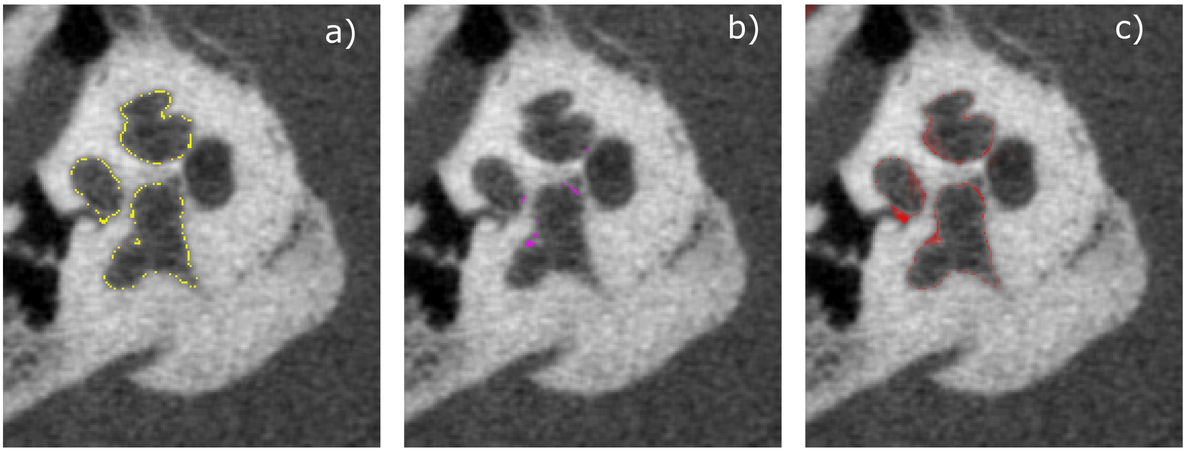

Supplementary Figure 11: False positive (a), false negative (b) error and supersamples standard deviation (c) maps for an automated prediction for a held-out test dataset element (dataset B). The error maps were computed using the manually annotation as ground truth labels. The main error mass of the automated prediction is located on the surface of the inner ear volume where high radiodensity gradients indicate a transition from osseous to cavernous structures and on soft-tissue nerve and membrane sites, where only a small contrast gradient between foreground and background volume exists.

S.3 Training Procedure Details

The experiments were conducted on two hosts. The first host was a workstation machine with an AMD Ryzen 3700X CPU, 64 GB RAM and a nVidia 2080 Ti (TU102) GPU with 11264 MB GDDR6 VRAM. The second host consisted of an AMD Epyc 7452 CPU, 2 TB RAM and a nVidia A100 SXM4 (GA100) with 40 GB HBM2e VRAM. Since the training data volumes, the network parameters and the gradients obligatorily reside on the GPU, mixed precision training [7] and gradient scaling [14] was essential to alleviate the large memory load incurred by the 3D framework and is subsequently used for all experiments. The parameters were fit in a empirical risk minimization strategy with the `AdamW` gradient descent optimization algorithm [4] using weight decay[6] as a regularization strategy. For the default setting, we adjusted the weight decay regularization coefficient to $\lambda = 2.5 \times 10^{-3}$ and the gradient running average coefficients to $\beta = (0.9, 0.997)$. A training data batch always consisted of two

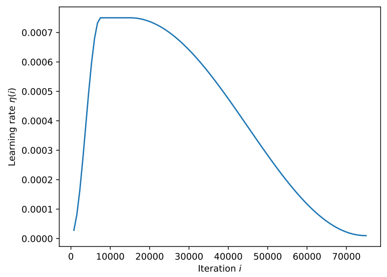

(a) The one-cycle learning rate η schedule where $\eta = \eta(i)$ is a function of the iteration i. The three phases warmup, plateau and anneal occupy fractions of 0.1, 0.1 and 0.8 of the total training steps respectively.

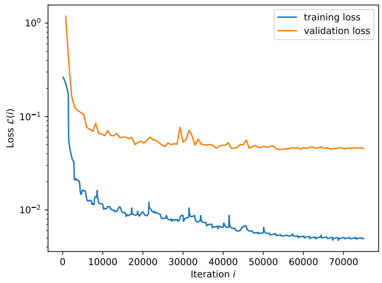

(b) The evolution of the training loss function value consisting of the cross entropy, soft Dice coefficient and mean squared loss terms. The combined loss is presented as a function of the iteration $\mathcal{L} = \mathcal{L}(i)$.

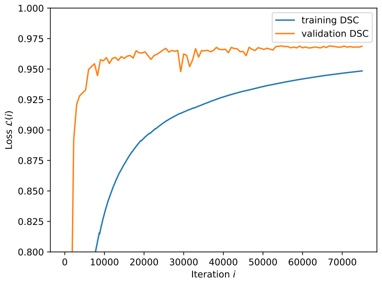

(c) Plot of the Dice coefficient computed as running average for training and on the validation dataset depending on the iteration.

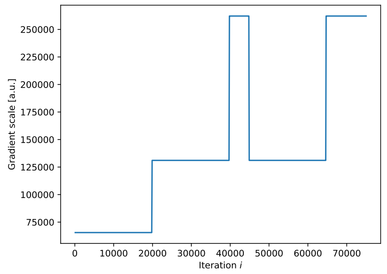

(d) Evolution of the gradient scaling factor. The gradient scaling factor is utilized for automated mixed precision training to mitigate underflow problems otherwise present with 16-bit floating point numbers.

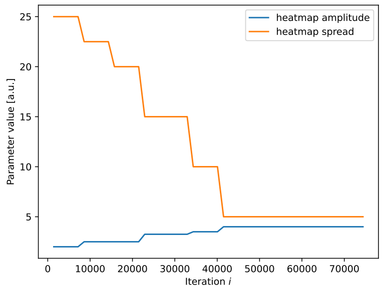

(e) The evolution of the heatmap amplitude and spread parameters for a training run with heatmap scheduling. At preset milestones, the amplitude is increased and the heatmap spread decreased.

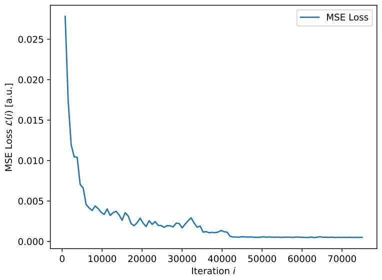

(f) Plot localization-specific mean squared error loss during a typical training run. The value is computed on the validation set.

Supplementary Figure 12: Evolution of the central training and validation parameters during a typical training iteration progression.

volume chunks, i.e. $B_s = 2$. To mitigate the problems induced by full stochastic gradient descent like noisy gradients or large gradient magnitudes from pathological chunks, we accumulated the gradients over $N_{\text{AccumIter}} = 10$ iterations in the default setting. The learning rate η was modified dynamically throughout the training in a single cycle policy. This policy raised the learning rate from an initial value to a maximum value (warmup phase). Then, the learning rate was held constant at this value (plateau phase) and was subsequently annealed to a small, final value (anneal phase). Both transient phases were traversed in a quarter-period cosine curve. Warmup phase, plateau phase and anneal phase occupied fractions of $(0.1, 0.1, 0.8)$ of the total steps, respectively.

S.4 Data Processing and Augmenting Transformations

In this section more detailed information about the setup of the data processing and augmenting transformations utilized during training and during test time is provided. The primary processing step was the resampling of the open-source dataset catalogue to the 99 µm isotropic voxel size that was defined by the training dataset A. A visual comparison of the multitude of raw voxel sizes utilized for this study is given in Sup. Fig. 13. Due to the open source datasets which provided its dataset instances cropped to a volume of interest and computational constraints, we evaluated all datasets on a volume of interest around the manually labeled inner ear structure. This strategy yielded input volumes with variable axis sizes for N_x, N_y and N_z, ranging approximately from 145^3 voxels (dataset W) to 280^3 voxels (datasets A and B). The U-Net core readily handled the variably sized input volumes due to the fully convolutional architecture. For the next processing step, we differentiated between in-house (A, B) and the different sub-clusters of open-source datasets (W, O, G) displayed in Fig. 1 of the main article. The more involved processing for the open-source datasets was based on the multiple insights. First, voxel-wise intensity

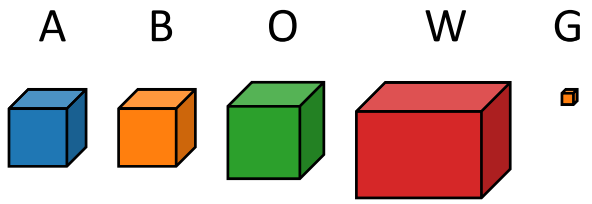

Supplementary Figure 13: Visual comparison of the raw data voxel sizes present in the different training and validation (A, 99 µm isotropic), test (B 99 µm isotropic) and open source (O 125 µm isotropic, W 150 µm × 150 µm × 200 µm, G {16.3 µm, 19.5 µm} isotropic) datasets used in this work.

analysis of dataset G and instances of O showed data features of extremal values not conforming to the Hounsfield scale used by the training dataset A (see also descriptive dataset statistics in section S.5). Secondly, further voxel-wise intensity analysis involving the foreground exhibited substantially altered contrast for the inner ear fluid volume in dataset G, O and W (specific to instances preserved in Formalin). Summarizingly, the described preservation methods, embedding in Formalin or Thiel for dataset W, serial multiple immersion in drying solutions and final embedding in epoxy resin for dataset O and complete desiccation with destructive effect on membraneous structures for dataset G induced input data shift that is generally not captured by the training data or mitigated by the augmentations outlined below. For both the in-house datasets and the open-source datasets we performed standardization and quantile clipping. For the transformations, the mean μ_{total} and standard deviation σ_{total} and quantiles $q_{0.01}$ and $q_{0.99}$ were computed using the full dataset (in-house datasets) or the various cluster groupings (open-source datasets). Voxel intensities smaller or larger than the quantile values were clipped to the respective value. Standardization amounted to voxel intensity computation according to the following mapping

$$\widetilde{v_i} = \frac{v_i - \mu_{\text{total}}}{\max(\epsilon, \sigma_{\text{total}})} \tag{1}$$

where $\epsilon = 1 \times 10^{-6}$ acted as a numerical stability safeguard. For the open-source datasets we additionally adopted a histogram-adjustment step to correct for the preservation method induced contrast shift detailed above and in the main manuscript. The goal was such that the average intensity, average foreground intensity and their respective standard deviations are commensurable after the application of the preprocessing scheme. The mapping given by eq. (2) defines this process.

$$\widetilde{V} = V + k_c \cdot (G_\sigma * F) \tag{2}$$

Here, $\widetilde{V}$ is the adjusted raw intensity volume, V the unadjusted volume, k_c is the adjustment strength, G_σ a 3D Gaussian kernel with standard deviation σ, F the foreground mask and $*$ denotes the convolution operation. The factor k_c was determined for the data cluster-wise to achieve commensurable histograms after clipping and standardizing with eq. (1). For the standard deviation of the kernel, $\sigma = 2$ was used for all experiments.

Chunk Processing The next pipeline step employed chunk-based processing with $(128 \times 128 \times 128)$-sized subvolumes and an isotropic stride of 25 voxels in x, y and z direction. After the forward pass through the model the chunks were automatically recomposed into the complete volume. This implies that voxels may be classified multiple times in variable spatial contexts due to their presence in multiple chunks. Precisely, this *implicit prediction supersampling* means that a voxel with spatial index i may be included in M different chunks and subsequently M forward passes with the voxel-wise prediction results $(p_i^1, ..., p_i^M)$. For the recomposition, we adopted a spatially weighted aggregation scheme, meaning that for the aggregation of multiple voxel-wise predictions the ones close to a subvolume chunk edge were down-weighted relative to central predictions. The final voxel-wise prediction was computed according to eq. (3) via weighted mean aggregation of the individual prediction results.

$$p_i^{\text{final}} = M^{-1} \sum_{m=1}^{M} w_i \cdot p_i^m \tag{3}$$

The weight was given by a constant matryoshka-like stencil that was fixed for all experiments. The specification of the stencil is given by $[5 : 0.10, 10 : 0.33, 20 : 0.80, 25 : 0.90]$, where the first integer number defines the orthogonal voxel distance interval from the edge of the subvolume and the secondary decimal number defines the relative weighting factor w_i for the voxel located in this matryoshka shell element.

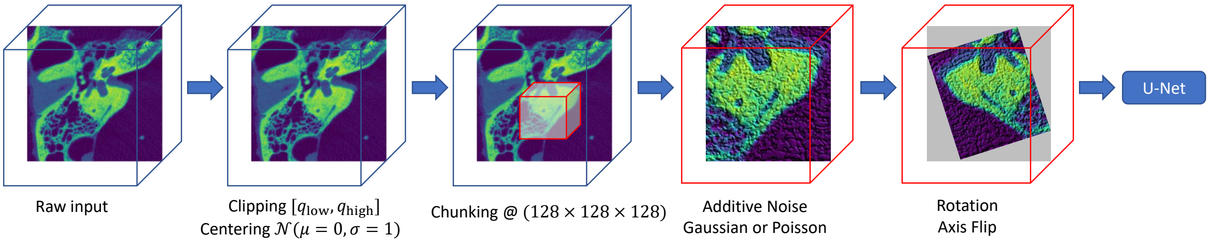

Supplementary Figure 14: Exemplary realization of the described transformation and augmentation pipeline. A selected volume chunk is first standardized by cropping the voxel values intensity-wise to the 0.01 and 0.99 quantile. Then the values are transformed to zero mean and unit variance. Subsequently, additive Gaussian or Poisson noise as well as random rotations and flips around all axes are applied stochastically.

Augmentations In addition to the deterministic and fixed processing outlined above, a number of contrast and intensity modifying augmentations were applied randomly during training. Their randomized application during an arbitrary training iteration was facilitated by comparing a randomly sampled number $\Theta \sim \mathcal{U}(0, 1)$ to a execution probability hyperparameter p_{exec} that was manually chosen for every such transformation. The augmenting transformation was only applied if $\Theta < p_{\text{exec}}$, otherwise the input data remained unchanged. A schematic overview of the process is displayed in Sup. Fig. 14. Purely spatial augmentations entailed the flipping of the volume about all axes, 90 °, 180 ° and 270° about all axes and interpolated free angle rotations with preset maximum amplitude about all axes. In the following, the transformations are described in detail.

Gaussian Noise This transformation was applied randomly with an execution probability p_{exec} of 0.33 for Aug_α and 0.5 for Aug_β. The voxel values in the volume $\mathbf{v}$ were altered by adding a random sampled noise vector η.

$$\widetilde{v_i} = v_i + \eta_i$$

The elements of the noise vector were sampled from a Gaussian distribution.

$$\eta_i \sim \mathcal{N}(0, \sigma_G)$$

The standard deviation σ_G of the Gaussian distribution was in turn sampled from a uniform distribution

$$\sigma_G \sim \mathcal{U}(a_G, b_G)$$

where the lower and upper boundaries a_G and b_G were manually set hyperparameters. For the experiments with protocol Aug_α, we used $a_G = 0.25$ and $b_G = 0.95$. Protocol Aug_α was generated by $a_G = 0.33$ and $b_G = 1.125$. A comparing view of a representative image slice in its raw, untransformed state and the augmented state is provided in Sup. Fig. 15.

Rotation The first step in this transformation entailed the random selection of a rotation plane defined by a pair of two axes. In the presented experiments, we utilized combinations of all three axes to define a rotation plane. The rotation itself was applied with randomly chosen rotation angles ϕ, where the rotation angle was sampled from a uniform distribution

$$\phi \sim \mathcal{U}(\phi_{\min}, \phi_{\max}) \tag{4}$$

where the lower and upper boundaries were manually set hyperparameters. We utilized $\phi_{\min} = -30°$ and $\phi_{\max} = 30°$ for protocol $\text{Aug}_\alpha$and $\phi_{\min} = -45°$ and $\phi_{\max} = 45°$ for protocol Aug_β. The rotation implementation `ndimage.rotate` of the Python package `scipy`[12] was used to compute the spline-interpolated rotated volume. For raw data, an interpolation spline of order of 2 was used, for the label data an interpolation spline of order 0 was used. The volume data was extended beyond its boundaries via reflection mode. In Sup. Fig. 15 a visual comparison of a non-transformed and rotated slice is shown. The effects of the reflect mode is visible in the lower right corner of the depiction.

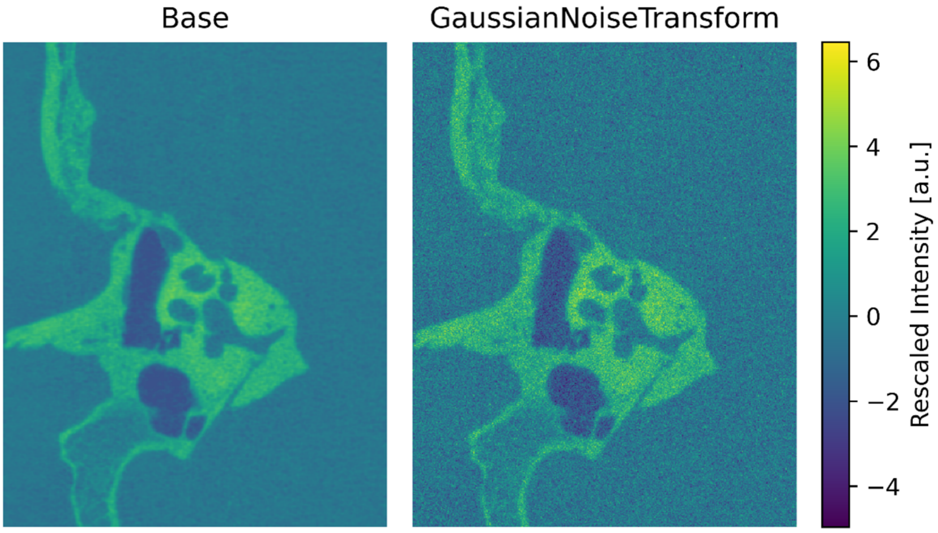

Supplementary Figure 15: Comparison of a representative slice with the original data on the left and the slice transformed by additive Gaussian noise on the right.

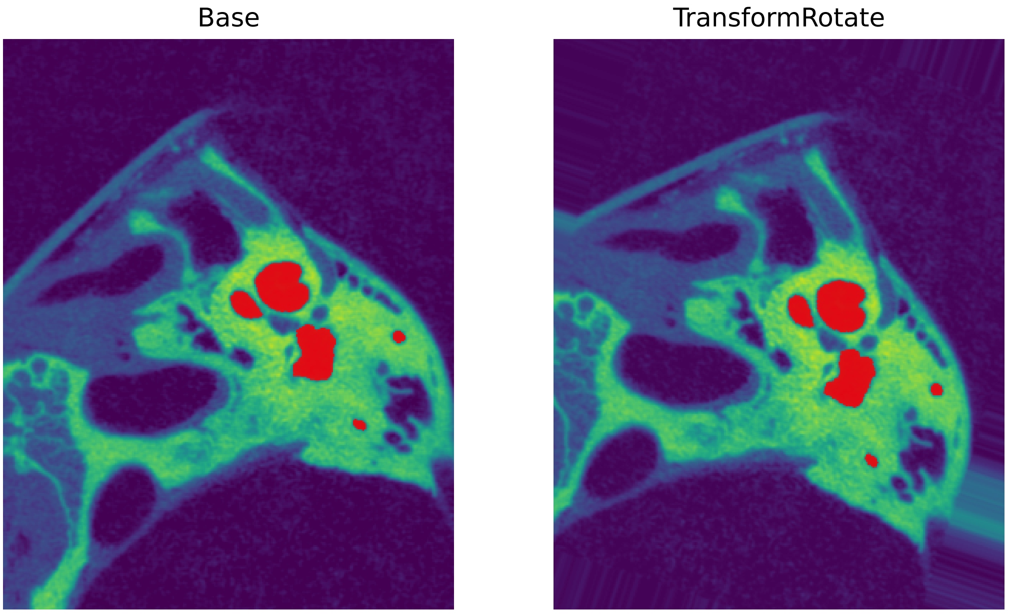

Supplementary Figure 16: Comparison of a representative slice with the basal unrotated raw data and label (red) on the left and the jointly rotated transformation result on the right.

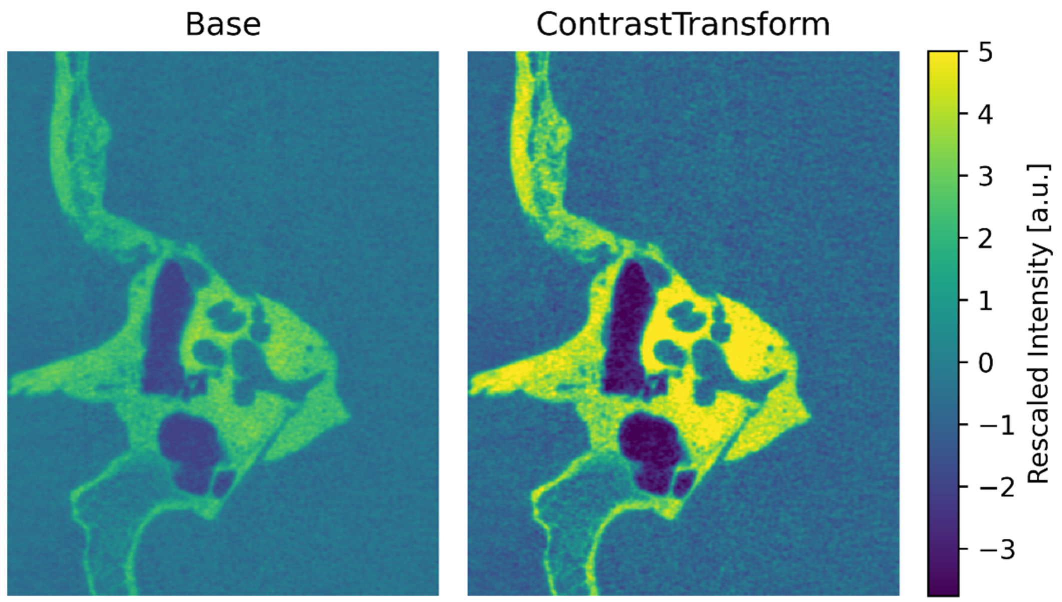

Supplementary Figure 17: Comparison of a representative slice with the original data on the left and the contrast-transformed slice on the right.

Contrast Transformation For this transformation, we set an execution probability p_{exec} of 0.25 for $\text{Aug}_\alpha$and 0.5 for Aug_β. The transformation encompassed a voxel-wise intensity value recomputation based on the dataset mean intensity value μ, the i-th voxel intensity value v_i and a randomly sampled contrast scaling factor k_{contrast}. The transformation is defined by the following equation

$$\widetilde{v_i} = \mu + k_{\text{contrast}} \cdot (v_i - \mu)$$

The contrast scaling factor was randomly sampled from a uniform distribution whose boundaries were defined by manually chosen hyperparameters.

$$k_{\text{contrast}} \sim \mathcal{U}(a_C, b_C)$$

This scaling factor random sampling occurred for every individual augmentation step. For the experiments with protocol Aug_α, the parameters were set to $a_C = 0.50$ and $b_C = 2.00$. For protocol Aug_β, the values $a_C = 0.5$ and $b_C = 2.125$ were used. As a last step, any voxel values exceeding a manually set pair of minimum and maximum values ($v_{\min} = -30, v_{\max} = +30$) were clipped to the respective boundary that was undercut or exceeded. In Sup. Fig. 17, a comparing view of a representative slice image in its raw and transformed state is provided.

Poisson Noise This transformation was applied randomly with an execution probability of $p_{\text{exec}} = 0.25$. Similar to the Gaussian noise transformation, the voxel values were modified by addition of a randomly sampled noise vector γ.

$$\widetilde{v_i} = v_i + \gamma_i$$

The elements of this noise vector were naturally sampled from a Poisson distribution.

$$\gamma_i \sim \text{Pois}(\lambda)$$

The distribution parameter λ was in turn sampled from a uniform distribution

$$\lambda \sim \mathcal{U}(a_P, b_P)$$

where the lower and upper boundaries a_P and b_P were manually set hyperparameters. For this work, the hyperparameter settings $a_P = 0.25$ and $b_P = 1.25$ were chosen. The difference between an un-augmented slice and the Poisson noise augmented slice is provided in Sup. Fig. 18.

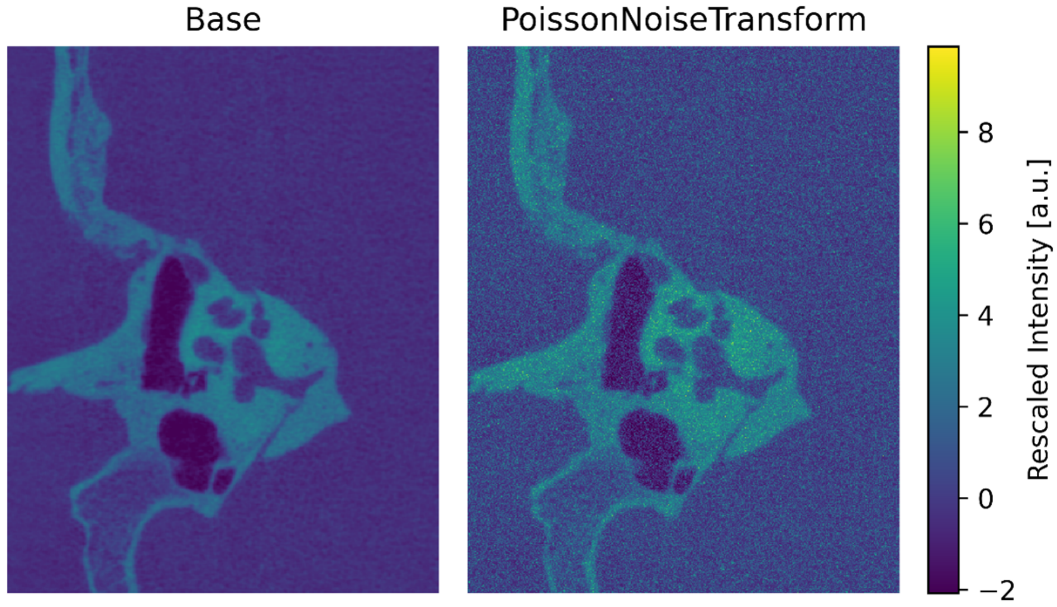

Supplementary Figure 18: Comparison of a representative slice with the original data on the left and the slice transformed by additive Poisson noise on the right.

Test Time Augmentations In addition to the training data augmentations we used test-time augmentations (TTA) for two purposes. On the in-house test dataset B, we used test-time augmentation to preliminarily gauge prediction uncertainty (see Sup. Fig. 11 panel c) while improvements of prediction performance were the second purpose on the open-source datasets. The utilized TTA protocol entailed the consecutive prediction for a volume chunk in its original state as read from the dataset instance and its nine augmented states generated from 90 °, 180 ° and 270° rotations around the three spatial axes. The resulting aggregate prediction for a voxel p_i^{final} with spatial index i undergoing N_{TTA} test-time augmentations was obtained by averaging the individual prediction supersamples. Similarly, the voxel-wise standard deviation σ_i and the volume-average standard deviation per voxel $\langle\sigma\rangle_v$ for a defined volume could be computed from the prediction supersamples as given in eq. (5).

$$p_i^{\text{final}} = \frac{1}{N_{\text{TTA}}} \sum_{a=1}^{N_{\text{TTA}}} p_i^a \qquad \sigma_i = \sqrt{\frac{1}{N_{\text{TTA}}} \sum_{a=1}^{N_{\text{TTA}}} \left(p_i^a - p_i^{\text{final}}\right)^2} \qquad \langle\sigma\rangle_v = \frac{1}{N_{\text{voxel}}} \sum_{i\in I} \sigma_i \tag{5}$$

Here, $N_{\text{voxel}} = \sum_{i\in I} 1$ denotes the number of voxels present in an arbitrary spatial volume with the index set I. For the presented results, a TTA scheme consisting of $N_{\text{TTA}} = 10$ prediction supersamples via rotation transformations was employed.

S.5 Descriptive Dataset Statistics

This section presents plots of descriptive statistics entailing dispersion parameters (minimum, maximum, standard deviation) as well as central tendency mean parameter of the various datasets. The descriptive statistical parameters were first computed per dataset instance and then visualized in aggregate form for every dataset as an element of the box plot. To support the cluster-wise analysis and processing of the open-source datasets, we provide separate evaluations for the Formalin (W_F) and Thiel (W_T) preserved instances of dataset W and the small (G_sFOV) and large (G_lFOV) field-of-view instances of dataset G. For some statistical parameters, detail-cropped box plots are presented when the resulting values exhibited a large range.

Interestingly, the open source datasets O, W and G contain minimum voxel intensity values below -1000. With respect to the basic definition of the Hounsfield scale given in eq. (6), this is an indication that the intensities of these datasets are not calibrated to the HU scale.

$$\text{HU} = 1000 \cdot \frac{\mu - \mu_{\text{water}}}{\mu_{\text{water}-\mu_{\text{air}}}} \tag{6}$$

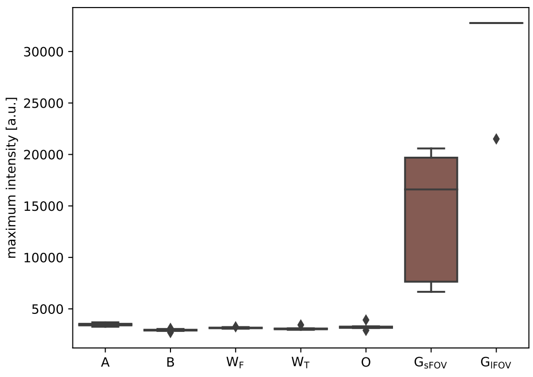

(a) Global maximum value statistics.

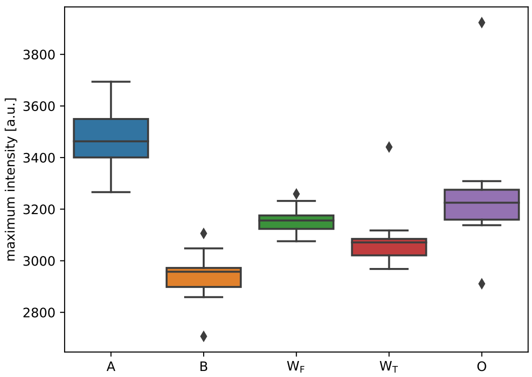

(b) Detail-cropped maximum value statistics.

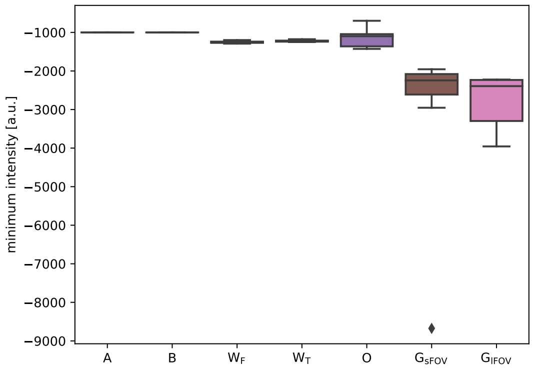

(c) Global minimum value statistics.

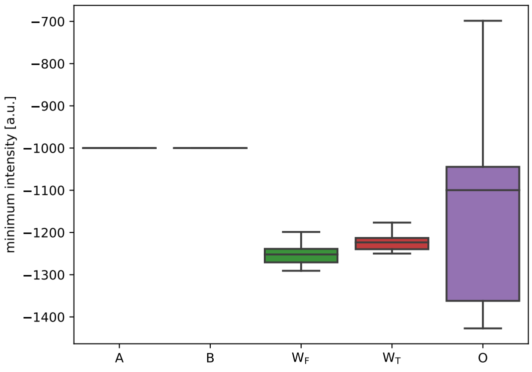

(d) Detail-cropped minimum value statistics.

Supplementary Figure 19: Overview of minimum and maximum intensity descriptive statistics of the in-house (A, B) and open source (O, W, G) datasets. Every parameter was computed per dataset instance. The corresponding aggregate information per dataset is visualized through the box plot items.

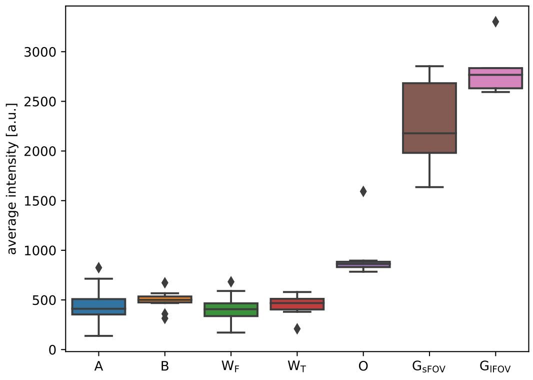

(a) Global average intensity statistics.

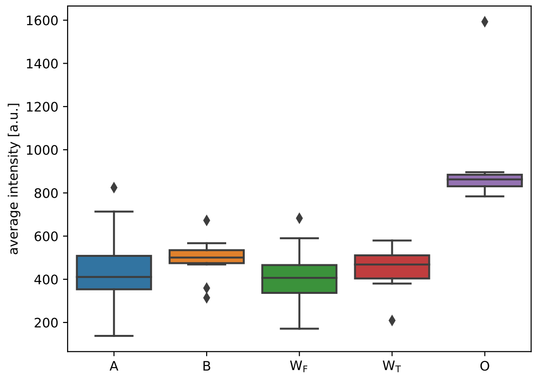

(b) Detail-cropped average intensity statistics.

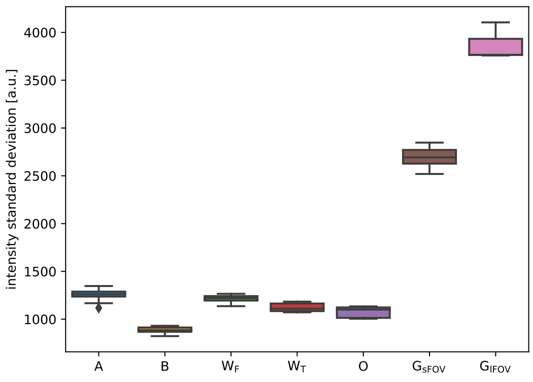

(c) Global intensity standard deviation statistics.

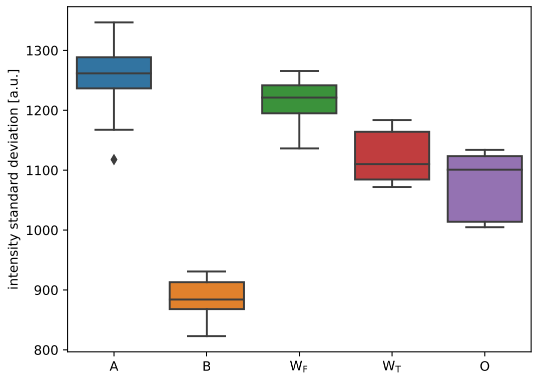

(d) Detail-cropped intensity standard deviation statistics.

Supplementary Figure 20: Overview of average intensity and corresponding standard deviation statistics of the in-house (A, B) and open source (O, W, G) datasets. Every parameter was computed per dataset instance. The corresponding aggregate information per dataset is visualized through the box plot items.

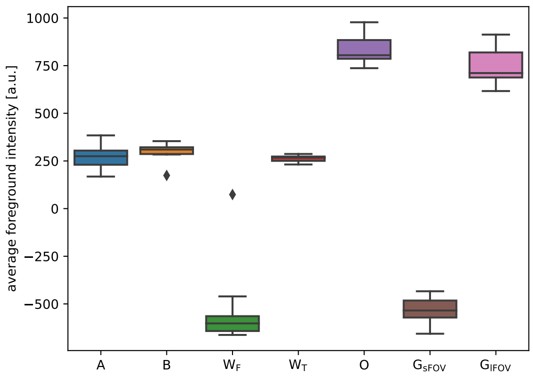

(a) Global average foreground intensity statistics per dataset.

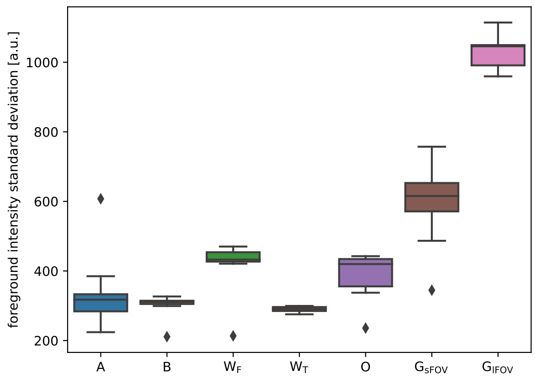

(b) Global average foreground intensity standard deviation statistics per dataset.

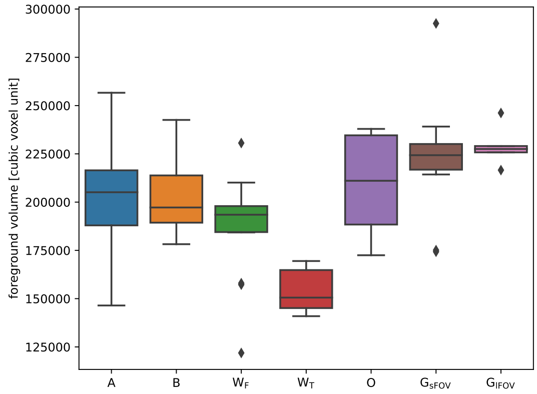

(c) Global inner ear foreground volume statistics per dataset.

Supplementary Figure 21: Overview of average intensity and corresponding standard deviation statistics of the in-house (A, B) and open source (O, W, G) datasets. Every parameter was computed per dataset instance. The corresponding aggregate information per dataset is visualized through the box plot items.

Additionally, we observed a large variation in the maximum intensity values, further underlining the necessity to perform the cluster-based processing of the open-source datasets.

S.6 Performance Metrics Description

General Metrics The performance of the presented algorithm and pipeline was evaluated with multiple metrics. In the following, the automated machine prediction composed of the semantic segmentation map and localization heatmap is denoted by $\mathbf{P} = (\mathbf{S}, \mathbf{H})$. Besides the machine prediction, the metric function required the manual label annotation $\mathbf{G}$ (ground truth segmentation maps and landmark coordinates) as inputs. In the context of crisp segmentation maps, the specified metrics can be computed via the true positive (TP), true negative (TN) and false negative (FN) classifications present in the segmentation map [10]. To produce a crisp segmentation prediction map where all voxels have a clear classification as inner ear or background $S_i^{\text{crisp}} \in \{0, 1\} \forall i \in I$, we applied a thresholding operation at the value 0.5 for the voxel-wise pseudo-probabilities $S_i \in [0, 1]$ that are supplied by the final sigmoid nonlinearity. Together with the ground truth maps G_i, the true positive, false positive and false negative voxel counts can be computed by:

$$\text{TP} = \sum_{i \in I} S_i^{\text{crisp}} \cdot G_i \qquad \text{FP} = \sum_{i \in I} S_i^{\text{crisp}} \cdot (1 - G_i) \qquad \text{FN} = \sum_{i \in I} (1 - S_i^{\text{crisp}}) \cdot G_i \tag{7}$$

The subsequently computed Dice score quantifies the overlap of the manual ground truth segmentation with the automated machine prediction by a scalar score varying between 0 (pessimum, no congruence) and 1 (optimum, perfect congruence). Using the above voxel count definitions, DSC and IoU as well as VS can be succinctly defined by

$$\text{DSC} = \frac{2\text{TP}}{2\text{TP} + \text{FP} + \text{FN}} \qquad \text{IoU} = \frac{\text{TP}}{\text{TP} + \text{FP} + \text{FN}} \qquad \text{VS} = 1 - \frac{|\text{FN} - \text{FP}|}{2 \cdot \text{TP} + \text{FP} + \text{FN}} \tag{8}$$

Other metrics like accuracy, sensitivity, precision and specificity can be trivially computed from the true positive, true negative, false positive and false negative voxel counts. They, however, provide limited insights due to the foreground-background imbalance that effects typical values close to saturation (i.e. 1.0). In addition to the overlap-based metrics laid out in eq. (8), we utilized the Hausdorff distance (HD) specified in eq. (9) as a contour-based evaluation criterion that takes the spatial structure of the segmentation into account.

$$\text{HD}(X, Y) = \max \left\{ \sup_{x \in X} \inf_{y \in Y} d(x, y), \sup_{y \in Y} \inf_{x \in X} d(x, y) \right\} \quad \text{where} \quad d(x, y) = \sqrt{\textstyle\sum_{j=1}^{3} (x_i - y_i)^2} \tag{9}$$

For this work, the Euclidean distance for the freely electable metric $d(x, y)$ was selected to compute the maximum contour distance of prediction and ground truth. The Euclidean distance was also utilized to compute the localization error of the landmarks (see eq. (10)).

$$d_{\text{euc}}^k = ||\mathbf{g}^k - \mathbf{r}^k||_2 \tag{10}$$

in the above equation, $||\cdot||_2$ is the L_2 norm, $\mathbf{g}^k$ is the ground truth coordinate vector of the k-th landmark and $\mathbf{r}^k$ is the coordinate prediction for the respective landmark as determined by the argmax operation on the emitted heatmap.

Loss function Similar to the general metrics, the loss components were computed using the U-Net prediction output $\mathbf{P}$, consisting of the segmentation map $\mathbf{S}$ and the heatmaps $\mathbf{H}$, and the corresponding manual ground truth information $\mathbf{G} \in \mathbb{R}^{n_x \times n_y \times n_z}$ as input. The manual ground truth information $\mathbf{G}$ either took the form of a ground truth label map for segmentation or ground truth heatmaps for localization. The default utilized loss consisted of the three elements Dice loss, cross entropy loss and mean squared error loss in the following definitions

$$\mathcal{L}_{\text{CE}} = -\frac{1}{|K_{\text{seg}}|} \sum_{k \in K_{\text{seg}}} \sum_{i \in I} G_i^k \log \left(S_i^k \right) \tag{11}$$

$$\mathcal{L}_{\text{sDSC}} = -\frac{2}{|K_{\text{seg}}|} \sum_{k \in K_{\text{seg}}} \frac{\sum_{i \in I} S_i^k G_i^k + c}{\sum_{i \in I} \left(S_i^k + G_i^k \right) + c} \tag{12}$$

$$\mathcal{L}_{\text{MSE}} = \frac{1}{|K_{\text{loc}}||I|} \sum_{k \in K_{\text{loc}}} \sum_{i \in I} (H_i^k - G_i^k)^2 \tag{13}$$

In the above definition, k denotes the class index for either the segmentation classes K_{seg} or the landmark classes K_{loc}, while i denotes the spatial index. The additive Laplace smoothing constant c in the Dice loss component inhibits singularities in case of prediction $\mathbf{S}^k$ and ground truth $\mathbf{G}^k$ volumes that only contain background (i.e. zero label) voxels. We used $c = 1$ throughout the experiments. The landmark loss term consisted of the mean squared error loss between the ground truth heatmap centered around the landmark coordinates $\mathbf{g}^k$ and the estimated heatmap $\mathbf{H}^k$. The ground truth heatmap for a given ground truth landmark coordinate vector $\mathbf{g}^k \in \mathbb{R}^3$ was produced by computing a 3D *Gaussian function* of heatmap values on the voxel grid. The center of the ground truth heatmap was naturally set to $\mathbf{g}^k$ with maximum voxel value of α and a spread of β. The values α and β were manually selected hyperparameters and part of our heatmap schedule.

$$\mathbf{G}^k = \alpha \cdot \exp\left(-\frac{(\mathbf{x} - \mathbf{g}^k)^2}{2\beta^2}\right) \tag{14}$$

S.7 Network Architecture Definitions

The BaseUNet architecture consisted of a feature map cascade of $\{32, 64, 128, 256, 320\}$, double convolutions with $(3 \times 3 \times 3)$ kernels, instance norm and LeakyReLU nonlinearity (negative slope parameter 0.025), downsampling via strided convolutions with $(2 \times 2 \times 2)$ kernel and isotropic stride 2, upsampling via transposed convolutions with a kernel size of $(2 \times 2 \times 2)$ and isotropic stride 2 and **no** attention gating. The segmentation head had sigmoid function as the final nonlinearity, the heatmap had had no final nonlinearity (linear output neurons for regression). The BaseUNet training protocol encompassed heatmap scheduling of subtype α, AdamW optimizer with $\lambda = 0.025$ and running average coefficients $\boldsymbol{\beta} = (0.9, 0.997)$, learning rate scheduling with trapezoidal form and warmup phase (10 % of N_{iter}), plateau phase (10 % of N_{iter})) and anneal phase (80 % of N_{iter}) with initial and final learning rate value $\eta = 1 \times 10^{-5}$ and peak learning rate $\eta = 7.5 \times 10^{-4}$. The base loss function was the linear combination of cross entropy loss eq. (11) and Dice loss eq. (12) for the segmentation head and mean squared error loss eq. (13) for the heatmap head. For single-task ablation experiments, only the respective head part and loss part were utilized. Deep supervision was part of the BaseUNet specification. The auxiliary outputs at the deeper levels t_1 to t_3 were produced by collapsing the channel-dimension via $(1 \times 1 \times 1)$ convolutions and subsequent trilinear upsampling to the input spatial dimension. The full loss value was computed by the linear combination of the loss value of the primary output and the auxiliary outputs. Deeper outputs were down-weighted as specified by the deep supervision loss coefficient sequence $\boldsymbol{f} = (1.0, 0.5, 0.33, 0.125)$.

Deep Supervision In addition to the default output maps at the highest decoder level, the network architecture was constructed to produce additional, secondary output maps[5] derived from terminals at lower decoder levels (t_1, t_2, t_3) in both heads. A terminal produced the secondary output map by first collapsing the input tensors along the channel dimension via $(1 \times 1 \times 1)$ convolutions and then spatially upsampling via trilinear interpolation. The structure and processing strategy of the described output terminals is visualized in Sup. Fig. 22. During training, "companion objectives" for lower encoder levels were formed by loss computation with the secondary output maps. The total loss of the head-specific task was computed as the weighted sum of the primary loss and all secondary loss values. The weighted-sum loss of the terminals for a specialized head utilized the hyperparameter factors $\boldsymbol{f} = (1.0, 0.5, 0.33, 0.125)$ for the primary level 0 and secondary levels 1 to 3, respectively. This deep supervision protocol acts as a regularization strategy and is expected to improve both performance and convergence [3] of the supervised learning of joint segmentation and localization.

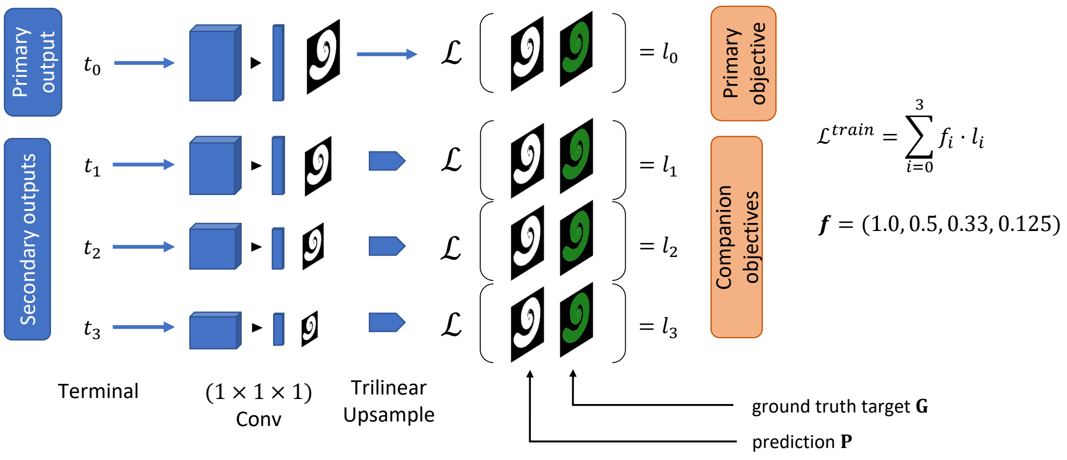

Supplementary Figure 22: Overview plot of the deep supervision terminal layout and companion objective processing chain. At the terminals t_0 to t_3, the primary output maps and secondary deep supervision output maps were extracted at training time. The secondary output maps were produced by a feature-map-wise contraction via $(1 \times 1 \times 1)$ convolution and subsequent trilinear upsampling. The total loss during the training phase was computed as a weighted sum of primary and secondary output maps with a manually selected set of level weights $\boldsymbol{f}$.

S.8 Implementation and Training of JSDNet

To provide a comparison against recently published state-of-the art methods, we looked towards works with focus on CMF-specific workloads, since no related works with that specific us case were available for temporal bone specific workloads. Since the formulation by Zhang et al. [13] is closely related to ours, we fully reimplemented the JSDNet framework in our PyTorch-based pipeline to provide another comparison data point. We reimplemented the JSDNet framework with its multi-staged training process from scratch, following the outlined architecture and training process description as closely as possible. For procedural information like learning rate and number of steps per training stage that were not specified in the study, we performed a cursory hyperparameter search and adopted a gradient step budget of 75000 iterations (similar to our base training process) that we distributed equally across the three stages, resulting in 25000 steps per stage:

- stage (1) training displacement map network FC1
- stage (2) training JSD with gradient flow to subnet FC1 disabled
- stage (3) training JSD with gradient flow through the full network

We modified three aspects of the original architecture by introducing instance norm and adaptive optimizer ADAM with learning rate scheduling instead of the originally specified stochastic gradient descent. The learning rate schedule encompassed trapezoidal warmup with cosine-formed flanks from $\eta = 5 \times 10^{-5}$ (initial and final value) and a peak of $\eta = 1 \times 10^{-4}$. Without the described adjustments, i.e. for SGD without normalization layers, we either observed slow or no convergence for JSDNet. Other key features, i.e. channel cascade sizes, max pooling and transposed convolutions layers as well as the cross entropy loss function remained true to the original formulation. Training subvolume chunk size was $(96 \times 96 \times 96)$ voxels. As described in the original work, inference was done with chunk size $(128 \times 128 \times 128)$ and weighted aggregation. We performed the training of JSDNet both without data augmentation (as specified in the original paper) and with our own default augmentation protocol (Aug_α).

References

[1] Sergey Ioffe and Christian Szegedy. "Batch Normalization: Accelerating Deep Network Training by Reducing Internal Covariate Shift". Mar. 2, 2015. arXiv: 1502.03167 [cs].

[2] Shruti Jadon. "A Survey of Loss Functions for Semantic Segmentation". In: *2020 IEEE Conference on Computational Intelligence in Bioinformatics and Computational Biology (CIBCB)* (Oct. 27, 2020), pp. 1–7. DOI: 10.1109/CIBCB48159.2020.9277638. arXiv: 2006.14822.

[3] Baris Kayalibay, Grady Jensen, and Patrick van der Smagt. "CNN-based Segmentation of Medical Imaging Data". July 25, 2017. arXiv: 1701.03056 [cs].

[4] Diederik P. Kingma and Jimmy Ba. "Adam: A Method for Stochastic Optimization". Jan. 29, 2017. arXiv: 1412.6980 [cs].

[5] Chen-Yu Lee et al. "Deeply-Supervised Nets". Sept. 25, 2014. arXiv: 1409.5185 [cs, stat].

[6] Ilya Loshchilov and Frank Hutter. "Decoupled Weight Decay Regularization". Jan. 4, 2019. arXiv: 1711.05101 [cs, math].

[7] Paulius Micikevicius et al. "Mixed Precision Training". Feb. 15, 2018. arXiv: 1710.03740 [cs, stat].

[8] Fausto Milletari, Nassir Navab, and Seyed-Ahmad Ahmadi. "V-Net: Fully Convolutional Neural Networks for Volumetric Medical Image Segmentation". In: *2016 Fourth International Conference on 3D Vision (3DV)*. 2016 Fourth International Conference on 3D Vision (3DV). Stanford, CA, USA: IEEE, Oct. 2016, pp. 565–571. ISBN: 978-1-5090-5407-7. DOI: 10.1109/3DV.2016.79.

[9] Ozan Oktay et al. "Attention U-Net: Learning Where to Look for the Pancreas". In: (Apr. 11, 2018).

[10] Abdel Aziz Taha and Allan Hanbury. "Metrics for Evaluating 3D Medical Image Segmentation: Analysis, Selection, and Tool". In: *BMC Medical Imaging* 15.1 (Dec. 2015), p. 29. ISSN: 1471-2342. DOI: 10.1186/s12880-015-0068-x.

[11] Dmitry Ulyanov, Andrea Vedaldi, and Victor Lempitsky. "Instance Normalization: The Missing Ingredient for Fast Stylization". Nov. 6, 2017. arXiv: 1607.08022 [cs].

[12] Pauli Virtanen et al. "SciPy 1.0: Fundamental Algorithms for Scientific Computing in Python". In: *Nature Methods* 17.3 (Mar. 2020), pp. 261–272. ISSN: 1548-7091, 1548-7105. DOI: 10.1038/s41592-019-0686-2.

[13] Jun Zhang et al. "Context-Guided Fully Convolutional Networks for Joint Craniomaxillofacial Bone Segmentation and Landmark Digitization". In: *Medical Image Analysis* 60 (Feb. 2020), p. 101621. ISSN: 13618415. DOI: 10.1016/j.media.2019.101621.

[14] Ruizhe Zhao et al. "Reducing Underflow in Mixed Precision Training by Gradient Scaling". In: *Proceedings of the Twenty-Ninth International Joint Conference on Artificial Intelligence, IJCAI-20*. Ed. by Christian Bessiere. International Joint Conferences on Artificial Intelligence Organization, July 2020, pp. 2922–2928. DOI: 10.24963/ijcai.2020/404.
